# Can terminators be used as insulators into yeast synthetic gene circuits?

**DOI:** 10.1186/s13036-016-0040-5

**Published:** 2016-12-16

**Authors:** Wenjiang Song, Jing Li, Qiang Liang, Mario Andrea Marchisio

**Affiliations:** School of Life Science and Technology, Harbin Institute of Technology, 2 Yikuang Street, Nan Gang District, Harbin, 150080 People’s Republic of China

**Keywords:** Terminator, Efficiency element, TATA box, Insulation, *S. cerevisiae*

## Abstract

**Background:**

In bacteria, transcription units can be insulated by placing a terminator in front of a promoter. In this way promoter leakage due to the read-through from an upstream gene or RNA polymerase unspecific binding to the DNA is, in principle, removed. Differently from bacterial terminators, yeast *S. cerevisiae* terminators contain a hexamer sequence, the efficiency element, that strongly resembles the eukaryotic TATA box i.e. the promoter sequence recognized and bound by RNA polymerase II.

**Results:**

By placing different yeast terminators (natural and synthetic) in front of the CYC1 yeast constitutive promoter stripped of every upstream activating sequences and TATA boxes, we verified that the efficiency element is able to bind RNA polymerase II, hence working as a TATA box. Moreover, terminators put in front of strong and medium-strength constitutive yeast promoters cause a non-negligible decrease in the promoter transcriptional activity.

**Conclusions:**

Our data suggests that RNA polymerase II molecules upon binding the insulator efficiency element interfere with protein expression by competing either with activator proteins at the promoter enhancers or other RNA polymerase II molecules targeting the TATA box. Hence, it seems preferable to avoid the insulation of non-weak promoters when building synthetic gene circuit in yeast *S. cerevisiae*.

**Electronic supplementary material:**

The online version of this article (doi:10.1186/s13036-016-0040-5) contains supplementary material, which is available to authorized users.

## Background

One of Synthetic Biology goals is the creation of standards that permit the exchange of basic DNA parts among labs and the faithful reconstruction of synthetic gene circuits. A commonly accepted way to characterize biological parts has not been established yet, although some have been proposed [1] and others are under development [2]. In general, a synthetic gene circuit can be represented as a network of transcription units that interact via the exchange of proteins or RNA molecules [3]. In eukaryotes a transcription unit is made of three parts: promoter, coding region (CDS), and terminator. Mathematical models often do not take into account the presence of CDS and terminator and identify a whole transcription unit with its promoter. Phenomenological models are preferred to mechanistic ones since they lump all the transcription regulation mechanisms into Hill functions that require the knowledge of two parameters: the Hill cooperativity coefficient (*n*) and the Hill half activation (or repression) constant (*K*_*H*_) [4]. In this framework, the dynamics of a generic mRNA *m* transcribed under a promoter *p* obeys the following ordinary differential equation

1 $$ \frac{dm}{dt} = k_{lk} + k_{tr} p \frac{\left(\frac{T}{K_{H}}\right)^{n \cdot c}}{1+\left(\frac{T}{K_{H}}\right)^{n}} - k_{d} m \,\,\text{,}   $$

where *k*_*lk*_ is the mRNA production rate constant due to promoter leakage, *k*_*tr*_ is the transcription initiation rate, *T* represents a transcription factor that binds *p* and regulates *m* synthesis, *k*_*d*_ is *m* decay rate, and *c* is a coefficient equal to 1 if *T* is an activator or 0 when *T* is a repressor. In principle, the leakage term can be omitted if promoter *p* is repressed since the Hill function equals zero only for an infinite amount of repressor *T*, whereas it has to be present in case of transcription activation. In this scenario, the leakage is entirely due to RNA polymerase II binding to a promoter in an inactive configuration i.e. either already occupied by repressor proteins or in absence of any activator that can help RNA polymerase II recruitment. However, leakage effects can be due also to RNA polymerase II moving along the DNA after a *read-through* from an adjacent terminator or an unspecific binding upstream a promoter TATA box–the entry point for RNA Polymerase II along a promoter sequence [5]. Therefore, even constitutive promoters, which can be regarded as always active, can be affected by leakage. Thus, in order to quantify the strength of a constitutive promoter properly, one should *insulate* the promoter, perhaps with the insertion of a terminator upstream its sequence [6].

Yeast terminators are characterized by three specific small sequences: the efficiency element, the positioning element, and the poly(A) site [7]. The efficiency element plays an important role in 3’ end formation. Its length and nucleotide composition varies from terminator to terminator. The strongest termination signal corresponds to the efficiency element TATATA [8]. RNA polymerase II can bind a promoter and start transcription in absence of enhancers [9] and promoter strength is highly dependent on its TATA box [10]. The sequences TATAAA [10] and TATAAAA [11] are the strongest TATA boxes in yeast *S. cerevisiae* and mammalian cells. However, the TATATA hexamer, i.e. the strongest terminator efficiency element, is also reported to be a strong TATA box [10]. Therefore, insulating a promoter with a terminator that contains the efficiency element TATATA corresponds to place a strong TATA box in front of a promoter.

In this work we show that the TATATA and other efficiency elements can indeed work as TATA boxes since they are able to recruit RNA polymerase II to the DNA and lead the transcription of a green fluorescence protein. This was proved with the construction of new synthetic promoters where a native or a synthetic terminator was placed in front of the yeast CYC1 promoter stripped of its two upstream activating sequences (UAS) and its three TATA boxes [12, 13]. As a consequence of their ability to bring RNA polymerase II to the DNA, terminators can disturb the transcriptional activity of a promoter if employed as insulators. All terminators used in this work decreased the expression of a green fluorescent protein when placed in front of the strong yeast GPD promoter [14]. Furthermore, the same terminator (from the DEG1 yeast gene [15]) showed different degrees of transcription down-regulation when placed in front of promoters of diverse strength. Hence, RNA polymerase II binding the insulator efficiency element interferes with activator proteins binding their enhancers and/or other RNA polymerase molecules that initiate transcription at the promoter TATA box. We show that a short distance between the efficiency element and the promoter UAS decreases protein expression drastically. However, insulator negative effects on transcription are still manifest when the efficiency element and the promoter UAS are considerably far away from each other. We also show that the strength of the efficiency element as a TATA box is another determinant factor of a decrease in protein expression. Moreover, we argue that DNA bending due to strong activators can foster the competition among RNA polymerases and the activator themselves to get access to the DNA. The experimental data illustrated in the next section allows us to point out when, in the construction of yeast synthetic gene circuits, promoter insulation should be avoided and when, in contrast, it is less likely to influence protein synthesis and, therefore, the performance of a whole circuit.

## Results and discussion

### Building new synthetic promoters by using terminator sequences

**Fig. 1:**
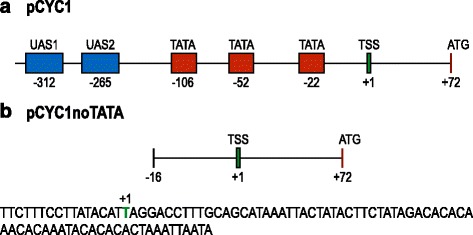
CYC1 promoters. **a** Scheme of the yeast CYC1 promoter as reported in [12, 13]. **b** Scheme and sequence of the pCYC1noTATA promoter used in this work. The thymine in green (+1 position) represents the promoter transcription start site (TSS)

In order to check if a terminator containing a TATA-box-like efficiency element can recruit RNA polymerase II molecules and initiate mRNA transcription, we constructed four synthetic promoters made of a terminator placed in front of the the yeast CYC1 promoter stripped of its two upstream activating sequences (UAS) and its three TATA boxes [12, 13]. We refer to this weak promoter as pCYC1noTATA (see Fig. 1).

**Fig. 2:**
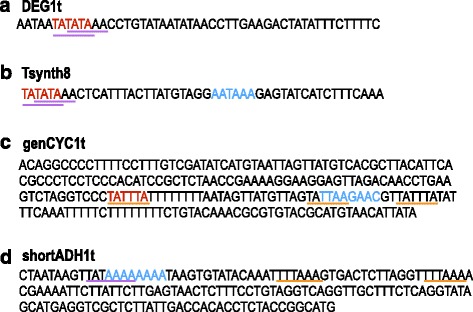
Terminators used to construct synthetic promoters. Sequences in *red* represent the efficiency elements, the ones in *blue* are the positioning elements (DEG1 positioning element is not known). Sequences underlined in *purple* are strong TATA-box motifs, the ones underlined in *orange* are weak TATA-box motifs

Among the terminators we chose, two contains the TATATA efficiency elements. They are the yeast DEG1 [15] (DEG1t) and the synthetic Tsynth8 [16] terminators. The latter, in particular, was chosen since it was described as one of the most efficient, among a collection of 30 synthetic terminators, to block RNA polymerase II read-through between two adjacent transcription units expressing different fluorescent proteins. In both DEG1t and Tsynth8 the efficiency element is followed by two adenines giving raise to the sequence TATAAA, the strongest eukaryotic TATA box (see Fig. 2 a–b). Besides, we chose the genomic CYC1 terminator (genCYC1t), whose weaker efficiency element (TATTTA) corresponds to a weak TATA box when yeast cells are grown in glucose medium (our case) [10, 17]. Moreover, inside genCYC1t another TATTTA sequence and a second TATA motif–TATTAA, classified as weak [10, 17]–can be found 34 and 22 nucleotides downstream the efficiency element, respectively (see Fig. 2 c). The last terminator we took into account is a shorter version of the yeast ADH1 terminator (shortADH1t–the sequence is shown in Fig. 2 d). Here, the efficiency element is missing but three possible TATA boxes are present: the strong TATAAAA and the weak TTTAAA [10, 17] at two different positions.

**Fig. 3:**
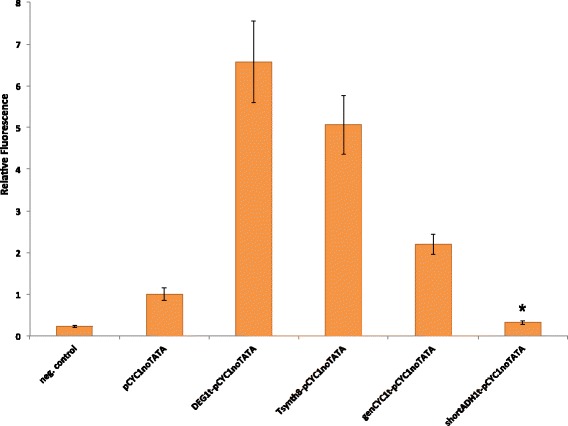
Characterization of synthetic promoters made of a terminator placed in front of pCYC1noTATA. Fluorescence levels are normalized with respect to the fluorescent signal produced by the bare pCYC1noTATA. The negative control (background relative fluorescence) is also shown. The strong TATA boxes into DEG1t and Tsynth8 cause, respectively, a 6.6 and 5.1 fold increase in pCYC1noTATA fluorescence level. The weaker TATA box belonging to the genCYC1t has a lower effect (2.2 folds increase). The * symbol indicates a statistically significant difference with respect to the fluorescence level of the negative control

We characterized the strength of our promoters via fluorescence measurements (FACS, see “Methods”). Every promoter leads the production of a yeast enhanced green fluorescent protein (yEGFP [18]). Our results (see Fig. 3) clearly show that the efficiency element inside yeast terminators is recognized as a TATA box by RNA polymerase II. The strength of our new synthetic promoters depends both on the type of TATA box inside the terminator and its distance from the transcription start site (TSS) into the pCYC1noTATA (see Table 1). A TATA box in yeast *S. cerevisiae* promoters is able to activate TSSes that lie from 40 up to 120 nucleotides downstream [12, 19]. DEG1t- and Tsynth8-containing promoters outperforms the other two synthetic constructs due to the presence of a strong TATA box along their sequences that is just less than 60 nucleotides upstream the TSS. Here it should also be noted that the minimal CYC1promoter sequence (pCYC1min–starting at position −72 with respect to the TSS) shows a distance of 46 nucleotides between its TATA box (TATATA) and the TSS. This configuration is close to the one of DEG1-pCYC1noTATA and, indeed, the two promoters produce a very similar fluorescence level (see Additional file 1). The strong TATA box at the beginning of the shortADH1t is too far from the TSS and therefore unable to activate it; the TATTTA element into genCYC1t, as expected, appears to be rather weak. The shortADH1t-pCYC1noTATA is the weakest of our synthetic promoters and its fluorescence is only less than 2 arbitrary units above the background one measured on the negative control strain (byMM2, which does not contain any synthetic artifacts).

**Table 1:** List of our new synthetic promoters with full specification of their TATA boxes (nt stands for nucleotides)

Promoter	TATA box	Kind	Distance from the TSS (nt)
DEG1t-pCYC1noTATA	TATAAA	Strong	53
Tsynth8-pCYC1noTATA	TATAAA	Strong	57
genCYC1t-pCYC1noTATA	TATTTA	Weak	112
	TATTAA	Weak	84
	TATTTA	Weak	72
shortADH1t-pCYC1noTATA	TATAAAA	Strong	160
	TTTAAA	Weak	135
	TTTAAA	Weak	116

Interestingly, pCYC1noTATA alone gives a fluorescence amount that, although low (18.5 Arbitrary Units–AU), is clearly above both the background (4.3 AU) and the shortADH1t-pCYC1noTATA (5.9 AU) fluorescence level. If we regard shortADH1t-pCYC1noTATA as an insulated pCYC1noTATA, we can conclude that leakage effects due to unspecific binding of RNA polymerase II upstream the pCYC1noTATA sequence account only for few (about 12) arbitrary units of fluorescence (with the machine setup we chose for our measurements). Therefore, they are negligible for strong promoters such as pGPD, whose average fluorescence level is about 612 AU.

**Fig. 4:**
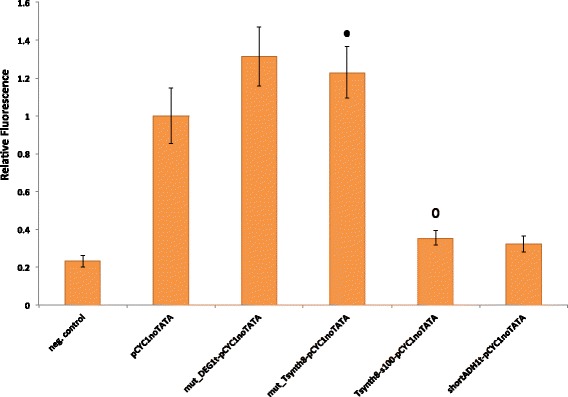
Mutational study on DEG1t-pCYCnoTATA and Tsynth8-pCYC1noTATA. Mutations on the efficiency element of DEG1t (mut_DEG1t-pCYC1noTATA) and Tsynth8 (mut_Tsynth8-pCYC1noTATA) make it no longer recognized by RNA polymerase II molecules as a TATA-box with a consequent decrease in fluorescent expression. The insertion of a 100-nucleotide-long spacer between Tsynth8 and pCYC1noTATA extends the distance between the TATA-box-like efficiency element and the TSS up to 157 nucletides. This prevent any TSS activation from the TATAAA motifs along Tsynth8. Fluorescence level are normalized to the one corresponding to pCYC1noTATA. The ∙ symbol indicates no statistically significant difference with respect to pCYC1noTATA fluorescence level, whereas the ∘ symbol indicates no statistically significant difference with respect to shortADH1t-pCYC1noTATA fluorescence level

In order to further ensure the role of the terminator efficiency element as an RNA polymerase II binding site, we mutated the TATATA sequence inside DEG1t-pCYC1noTATA and Tsynth8-pCYC1noTATA into GAGATA. As shown in Fig. 4, this double point mutation makes the average fluorescence level of both our synthetic promoters drop to, approximately, the one of the bare pCYC1noTATA. As a further test, we placed a sequence of 100 nucleotides (s100) between the Tsynth8 terminator and the pCYC1noTATA to have a distance between the TATA box and the TSS longer than 120 nucleotides. The new resulting synthetic promoter Tsynth8-s100-pCYC1noTATA produces a very low fluorescence amount just higher than the negative control and comparable to the one corresponding to shortADH1t-pCYC1noTATA (see Fig. 4). This also supports our hypothesis that shortADH1t is a proper insulator for pCYC1noTATA.

### Insulating the strong GPD promoter

Transcription units into bacterial synthetic gene circuits can be insulated by placing a terminator in front of the promoter. Bacterial promoters contain, around position −10, a TATA-like hexamer recognized by RNA polymerase. However, bacterial terminators are very different from the eukaryotic ones: they have a palindromic region rich in guanines and cytosines followed by at least six thymines. Upon transcription, the G-C-rich sequence folds into an hairpin that slows down the motion of RNA polymerase. Taking advantage of the weak *U*−*A* bond between mRNA and DNA in the active site once the T-rich motif has been transcribed, RNA polymerase can escape from the DNA.

**Fig. 5:**
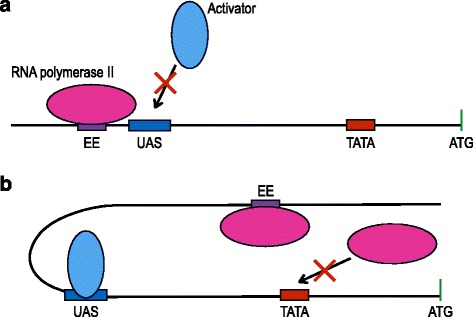
Possible scenarios for promoter competition induced by the presence of a terminator-insulator. **a** A strong insulator efficiency element (EE) is placed in proximity of the promoter upstream activating sequence. DNA steric occupancy by RNA polymerase II at the EE prevents activator binding at the UAS and recruitment of other RNA polymerase II molecules to the promoter TATA box. **b** DNA bending–here due to the presence of an activator at its UAS–puts the EE spatially close to the TATA box causing a competition among RNA polymerase II molecules to get access to the EE and the TATA box themselves. DNA bending might also put the EE near the UAS provoking a competition (or even a collision) between RNA polymerase II and activator molecules

As shown above, the efficiency element of yeast terminators is able to recruit RNA polymerase II due to its resemblance to a TATA box. Once placed in front of a constitutive yeast promoter, the efficiency element of a terminator is too far away from any TSS to increase gene transcription. However, it might interfere somehow either with promoter activation, by reducing the binding of activator proteins to the corresponding enhancers, or directly with the transcription initiation process. The latter circumstance can arise, for instance, if the terminator efficiency element gets spatially closer to the promoter TATA box because of the DNA bending provoked by an activator or the general transcription factor (see Fig. 5). As a result, promoter insulation would not just eliminate leakage effects but also reduce considerably the transcription initiation rate of the promoter itself.

**Fig. 6:**
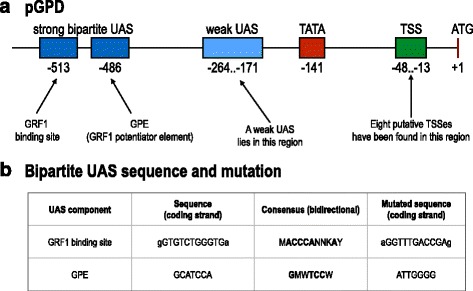
GPD promoter. **a** Schematic of pGPD structure. **b** Wild-type, consensus, and mutated sequence of the two components of the strong pGPD UAS. Notice that the UAS can be placed on either DNA strand. Low-case letters indicate mutated nucleotides just outside the GRF1 binding site

To test this hypothesis, we placed each of the four terminators described in Fig. 2 in front of the yeast GPD promoter [14] (pGPD) i.e. the strongest constitutive promoter in yeast *S. cerevisiae* [9] and measured, via FACS experiments, the fluorescence levels produced by these constructs. We consider, as GPD promoter sequence, the first 680 nucleotides upstream the start codon of the yeast GPD (also referred to as TDH3) gene. The GPD promoter is characterized by the presence of both a strong bipartite UAS starting at position −513 (beginning of the binding site of the GRF1 activator) and a weaker one located somewhere between position −264 and −171. Moreover, a strong TATA box (TATATAAA) lies between position −141 and −134. Here, all the positions are relative to the GPD start codon [14] (see Fig. 6 a). GRF1 activator is reported to cause DNA bending [20].

From the results on the terminator-pCYC1noTATA synthetic promoters, we concluded that leakage effects on constitutive strong promoters should be negligible. Therefore, if the four terminators we chose worked as pure insulators with no interference whatsoever with pGPD transcription initiation process, we would expect to see only a small difference between the fluorescence levels of the four terminator-pGPD constructs and the one of the wild-type pGPD. Roughly, a properly-insulated pGPD would express about 98% of the fluorescence measured on the wild-type pGPD.

**Fig. 7:**
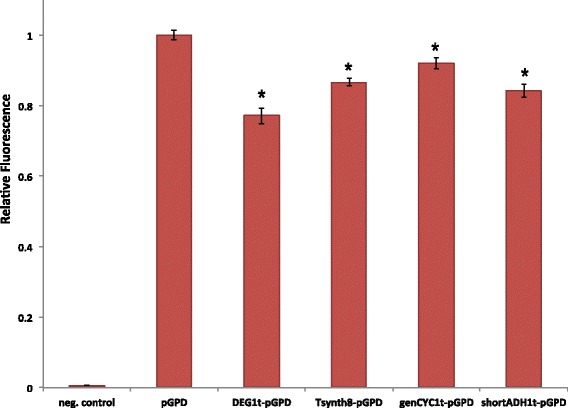
Fluorescence levels of synthetic promoters made of different terminators preceding the strong GPD promoter. All fluorescence levels are normalized with respect to the one of wild-type GPD promoter. The * symbol indicates a statistically significant difference with respect to pGPD fluorescence level. Among the four terminators, DEG1t causes the most considerable reduction in pGPD fluorescence level

The results in Fig. 7 show that each terminator determines a non-negligible reduction in the GPD promoter fluorescence level. The most consistent decrease is due to DEG1t, whose fluorescence level is only about 77% of the wild-type pGPD one. Tsynth8 and shortADH1t show a comparable rather strong effect (87% and 84%, respectively), whereas fluorescence reduction due to genCYC1t is lower (92% of the wild-type pGPD) but still too high to be due to leakage only. These results seem to confirm our hypothesis that a strong efficiency element in front of a constitutive promoter can reduce the promoter strength as a result of its capability to recruit RNA polymerase II molecules (see the Modeling section of the Additional file 1 for a theoretical representation of the DEG1t-pGPD system). According to this data, the strength of the TATA-boxes along the terminator sequences would play a more important role than their distance from the promoter UAS in reducing GFP expression from pGPD. This would explain the relatively low impact of genCYC1t on pGPD fluorescence level (no strong TATA boxes) and the similar results from shortADH1t and Tsynth8 despite the fact than the strong TATA box along Tsynth8 is over 100 nucleotides closer to the pGPD bipartite UAS than the one on the shortADH1t sequence (see Additional file 1). However, as we will show below, a short distance (less than 50 nucleotides) between the insulator efficiency element and the promoter UAS can have dramatic repercussions on fluorescence expression.

**Fig. 8:**
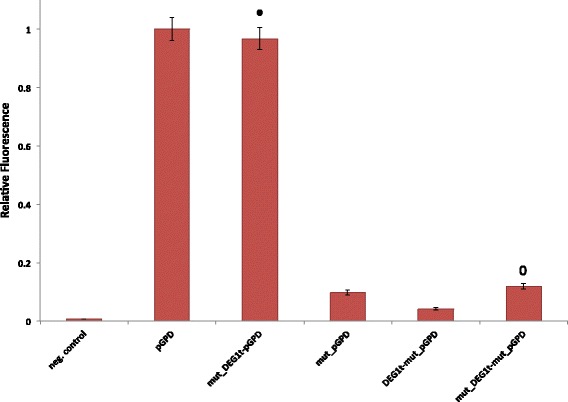
Mutational study on DEG1t-pGPD and DEG1t-mut_pGPD. Similarly to pCYC1noTATA, the fluorescence level of the wild-type pGPD is restored by removing, via double mutations, the TATA-box-like motif from the DEG1t efficiency element. Moreover, DEG1t is proved to be able to reduce the already rather low fluorescence expressed by mut_pGPD, where the sequence of the strong bipartite UAS has been deeply modified to prevent GRF1 binding. Therefore, RNA polymerase II molecules recruited by DEG1t are able to interfere also with far downstream transcription activation processes. By substituting DEG1t with mut_DEG1t, fluorescence grows back to the original level of mut_pGPD. All fluorescence levels are normalized with respect to the wild-type pGPD one. The ∙ symbol indicates no statistically significant difference with respect to pGPD fluorescence level, whereas the ∘ symbol indicates no statistically significant difference with respect to mut_pGPD fluorescence level

We focused on the sole DEG1t since it has the highest impact on pGPD transcriptional activity. We replaced, in front of pGPD, the wild-type DEG1t with the same mutated version we used with pCYC1noTATA (mut_DEG1t). As it is shown in Fig. 8, the double mutation on the efficiency element of DEG1t has the effect to restore the original pGPD fluorescence level. We then constructed a new version of pGPD (mut_pGPD) where the two components of its strong bipartite UAS have been mutated to prevent GFR1 binding (see Fig. 6 b). The fluorescence level of mut_pGPD is only about 10% of pGPD one. By placing DEG1t in front of mut_pGPD we detected a further decrease (over 50%) in fluorescence expression. Therefore, RNA polymerase II molecules, recruited at DEG1t efficiency element, are able to interfere even with the residual promoter activation process that takes place at the far pGPD weak UAS (see Fig. 6 a). By exchanging DEG1t with mut_DEG1t, the original fluorescence level of mut_pGPD is restored (see Fig. 8).

### Insulating different promoters

**Fig. 9:**
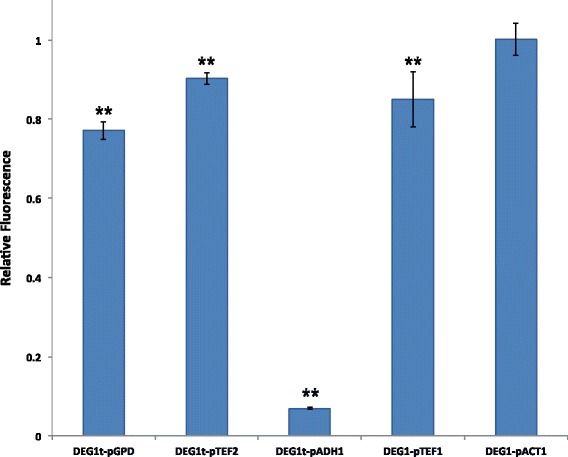
DEG1t as insulator of five different yeast constitutive promoters. DEG1t drives a decrease in the fluorescence level of strong and medium-strength promoters, whereas it seems not to spoil the transcriptional activity of the rather weak ACT1 promoter. Values shown in this Figure are the ratio between the fluorescence levels of the insulated promoters with respect to the ones of the non-insulated promoters. The ** symbol indicates a statistically significant difference between the fluorescence expressed by an insulated promoter with respect to the one corresponding to its wild-type configuration

So far, we have shown that the efficiency element of yeast terminators is able to recruit RNA polymerase II molecules due to its similarity to a TATA box. This prevent terminators–particularly DEG1t–from working as proper insulators when placed in front of the strong yeast GPD promoter since they disturb its transcriptional activity. We tried, then, to assess if DEG1t has a similar impact on the strength of other yeast promoters such as: TEF2 promoter [21] (pTEF2, about half as strong as pGPD), ADH1 promoter [22] (pADH1, approximately as strong as pTEF2), TEF1 promoter [23] (pTEF1, around 30% of pGPD strength), and ACT1 promoter [24] (pACT1, about 15% of pGPD strength–see Additional file 1). The structure of these four promoters–compared to pGPD one–is given in Table 2. DEG1t has a clear influence on yEGFP expression from pTEF1 and pTEF2 and even a dramatic one on pADH1, whereas no change was detected with pACT1 (see Fig. 9). pADH1 and pTEF2 share the same strength. However, pADH1 UAS is much closer than pTEF2 UAS to the DEG1t efficiency element. In the above analysis on pGPD insulated by different terminators we argued that the distance between the TATA-box-like motif on the insulators and the pGPD UAS was not determinant of fluorescence decrease. However, those distances were all above 200 nucleotides as it is the case of DEG1t-pTEF2. pADH1 UAS is only 43 nucleotides downstream the DEG1t efficiency element: this probably puts RNA polymerase II and the GCR1 activator in strong competition for promoter binding (conceptually not different from the one we engineered, in a previous work, to regulate transcription from a yeast synthetic promoter [25]). As a result, DEG1t-pADH1 shows a fluorescence level corresponding to about 7% of the one of the non-insulated pADH1. This

**Table 2:** Structures of the five yeast promoters we insulated with DEG1t

Promoter	Length	UAS	Distance DEG1t(TATAAA)-UAS (nt)	Activator	TATA box
pGPD	680	–513	205	GRF1	–141
pTEF2	645	–456	226	GRF1	–127
pADH1	700	–694	43	GCR1	–128
pTEF1	401	–324	114	GRF1	–120
pACT1	489	–410	116	GRF2	–196

UAS and TATA box position refers to the ATG start codon. Only the leftmost UASes in the promoter sequences are here considered

points out that RNA polymerase II should also have higher affinity to its binding site than the one GCR1 has towards its enhancer. No fluorescence reduction was observed on pACT1, the weakest among the promoters we considered in this analysis. This suggests that terminators might be used as proper insulators in front of promoters that are activated poorly. However, as we have seen above in the analysis of mut_pGPD (roughly 70% as strong as pACT1) also the transcriptional activity of weak (synthetic) promoters can be influenced by an insulator.

### Placing an insulator between two transcription units

**Fig. 10:**
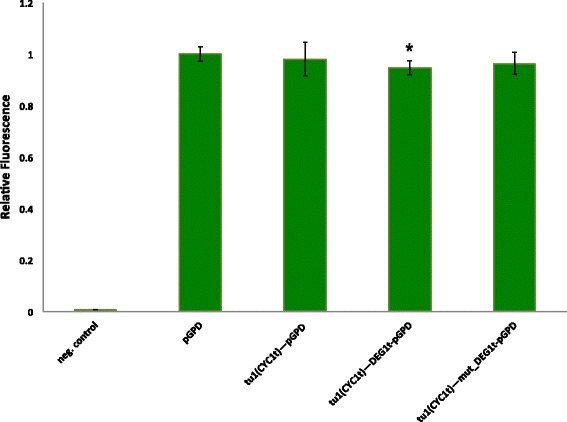
Green fluorescent protein expression by two-transcription-unit systems. DEG1t placed between two transcription units does not alter, in a considerable way, the production of GPF by the downstream transcription unit. This scenario is quite different from the one illustrated in Fig. 7. We think that two adjacent transcription units integrated into the same *S. cereviaese* genomic locus reduce DNA bending and, as a consequence, the competition between the RNA polymerase II molecules recruited by the insulator efficiency and other molecules targeting nearby DNA sequences. Each construct is labeled as tu1(CYC1) (i.e. the leftmost transcription unit that encodes for yomKate2 and ends with CYC1t) and the name of the insulator (if present) placed in front of the GPD promoter that drives yEGFP expression. Fluorescence levels are normalized with respect to the one expressed by pGPD on a single transcription unit. The * symbol indicates a statistically significant difference with respect to the wild-type pGPD fluorescence level

One of the reasons why *S. cerevisiae* is chosen as a chassis for synthetic gene circuits is the relative easiness of genome manipulation (genomic integration). One common technique exploits homologous recombination and auxotrophic selection. However, yeast strains are usually mutated only in a handful of auxotrophic markers. Therefore, the construction of complex circuits made of several interacting genes would require to place two or more transcription units inside a unique vector such that they are later on integrated into the same locus. Our previous analysis on single transcription units indicates that an insulator is likely to reduce the transcription efficiency of strong and medium-strength promoters considerably. What happens if we place an insulator between two transcription units? To answer this question we built a simple system where two adjacent transcription units express diverse fluorescent proteins (yomKate2–red fluorescence [26]–and the yEGFP) i.e. they are not interacting (a slightly similar analysis, though on more complex circuits, was recently presented in [27]). We inserted DEG1t and mut_DEG1t between the two transcription units (see Additional file 1 for the scheme of these constructs) and measured both green and red fluorescence expressed by these overall three systems. As a control, we used a single transcription unit producing either yEGFP or yomKate2. As we expected, yomKate2 synthesis is basically insensitive to any downstream constructs (see Additional file 1). However, GFP fluorescence level was not modified by an upstream transcription unit either. DEG1t, once placed between the two transcription units, provoked a reduction in GFP expression (95% of non-insulated pGPD), though very far from the strong one we reported above (77% of non-insulated pGPD). This small decrease disappeared after turning DEG1t into mut_DEG1t (see Fig. 10). As we mentioned previously, DNA bending can play an important role in the reduction of protein expression from insulated genes by enabling strong competition between RNA polymerase II molecules binding at the insulator efficiency element and either other RNA polymerase II molecules binding the promoter TATA box or activator proteins targeting their enhancer. We suppose that two adjacent transcription units integrated into the same locus reduce somehow the extent of DNA bending and make less probable a competition among RNA polymerase II and activator proteins. As a consequence, the presence of an insulator in front of a promoter becomes almost irrelevant for the expression of the downstream gene.

## Conclusions

Used as an insulator, a terminator should stop RNA polymerase II that bound to an unspecific site upstream the promoter sequence from reaching the promoter TATA box and start mRNA transcription. This leakage effect should be removed to characterize promoter strength properly and, therefore, improve synthetic gene circuit design. However, yeast terminators contain a hexamer, the efficiency element, that is in general very similar to a TATA box. In particular, the strongest efficiency element, TATATA, is identical to one of the strongest eukaryotic TATA boxes.

By fusing four different terminators in front of what we called pCYC1noTATA (i.e. the sequence of the yeast constitutive CYC1 promoter from which we removed all the UASes and TATA boxes) we proved that the efficiency elements are able to recruit RNA polymerase II molecules from the cell nucleus and lead to fluorescent protein expression. Moreover, we could estimate that the contribution of leakage effects to constitutive promoter transcriptional activity is very low.

When we placed the same terminators in front of the strongest yeast constitutive promoter, pGPD, fluorescence production dropped remarkably. This reduction could not be explained by the sole leakage removal. Hence, we concluded that the terminator efficiency elements interferes with transcription initiation by recruiting RNA polymerase II molecules.

Among the four terminators we used in this work, DEG1t proved to be the one with higher affinity towards RNA polymerase II. We insulated five yeast constitutive promoters of different strength with the DEG1 terminator. Only the weakest among these promoters (pACT1) turned out to be insensitive to the insulator presence, the other four underwent a non negligible reduction in transcription efficiency. In particular, ADH1 promoter fluorescence level dropped to the 7% of its original value. This effect was due to the short distance between the DEG1t efficiency element and pADH1 UAS (43 nucleotides only). RNA polymerase II binding the insulator efficiency element is, however, able to interfere with the promoter transcriptional activity also when a high distance separates the efficiency element from the promoter UAS. This was apparent from our experiments on a re-engineered GPD promoter where the strong bipartite UAS was almost completely mutated such that transcription was activated by a weaker UAS downstream. Once insulated with DEG1t, the fluorescence level of the mutated pGPD dropped by about one half, despite the fact that the distance between the efficiency element and the weak UAS was higher than 450 nucleotides.

Probably, DNA bending is a factor that enhances the interaction between RNA polymerase II binding the insulator efficiency element and either the activators targeting the enhancers or other RNA polymerase II binding the promoter TATA box. We argue that, by integrating two adjacent transcription units into the same locus of the *S. cerevisiae* genome, the effect of DNA bending is somehow lowered. This would explain why DEG1t, once inserted between two transcription units, caused only a marginal reduction in the expression of the downstream protein.

Overall, our results point out clearly that a single transcription unit containing a strong or medium-strength promoter should not be insulated with a terminator when integrated into the genome of yeast *S. cerevisiae*. Insulation, indeed, would provoke a reduction in protein expression that might have high repercussions on the performance of a whole synthetic gene circuit.

## Methods

### Plasmid construction

The yeast integrative shuttle-vector plasmid pRSII406 (Addgene-35442, a gift from Steven Haase) [28] was used as a backbone for the construction of every transcription unit. pGPD, pACT1, the CYC1 promoter (pCYC1), and the genomic CYC1 terminator (genCYC1t) were extracted from the yeast *S. cerevisiae* genome (strain FY1679-08A, see below) following the procedure in [29]. pCYC1 served as a template to PCR out the sequence of pCYC1noTATA and pCYC1min. pTEF2 was obtained from the MIT Registry part BBa_K801010, pTEF1 was extracted via PCR from pRS404-pTEF1-Ago1 (Addgene-22313, a gift from David Bartel), pADH1 was obtained from pHCA/GAL4(1-93).ER.VP16 [30] (courtesy of Picard lab, University of Geneva, Switzerland).

Every transcription unit expresses either the yomKate2 red fluorescent protein obtained from pFA6a-link-yomKate2-CaURA3 (Addgene-44878, a gift from Wendell Lim and Kurt Thorn) or the yeast enhanced green fluorescent protein (yEGFP) obtained from pRS31-glag [31] (courtesy of Hasty lab, University of California, San Diego, USA). A slightly different sequence (yEGFPgg) was used in the plasmids constructed with the MoClo method [32]. Here, the internal BsaI site was removed via silent mutation. The CYC1 terminator (CYC1t), placed at the end of every transcription unit, is described in [33] and is slightly different from genCYC1t [34]. We obtained it from pRS403-pGAL1-strongSC_GFP (Addgene-22316, a gift from David Bartel).

The ADH1 terminator used in this work (shortADH1t) was constructed by using, as a template, the MIT Registry part BBa_K801012. Insulated promoters were extended via PCR to be preceded by DEG1t (50 nt long), Tsynth8 (49 nt long) or their mutated version. The spacer s100 was taken from the bacterial *tetR* gene. The constructs genCYC1t-pCYC1noTATA, Tsynth8-s100-pCYC1noTATA, DEG1-pCYC1noTATA (for the MoClo assembly, see Additional file 1), and part of mut_pGPD were synthesized by GENEWIZ Inc. (Suzhou, China).

Plasmids were constructed either via isothermal assembly [35] or MoClo method [32]. For the MoClo assembly, we used the original universal 0-level acceptor vector pAGM9121 (Addgene-51833, a gift from Sylvestre Marillonnet) [36] and the 1-level acceptor vector ypL1F-1_406 that we constructed by adapting pRSII406 to host a transcription unit between the BpiI cutting sites TGCC and GCAA (for the original pL1F-1 see [32]). Primers for PCR were designed according to the chosen DNA assembly method.

Touchdown PCR was employed to select and amplify DNA sequences. DNA elution from agarose gel was carried out with the QIAGEN-28604 “DNA Elution kit”. Isothermal assembly required always one hour at 50 °C. In order to assemble 1-module plasmids the insert (0-modules) and our ypL1F-1_406 1-level acceptor vector were combined in 2:1 molar ratio and mixed with a master mix (1 *μ* *l* BsaI 20 units /*μ* *l*, NEB-R0535S; 2 *μ* *l* Cutsmart buffer NEB; 1 *μ* *l* T4 ligase 400 units /*μ* *l*, NEB-M0202S; 2 *μ* *l*10 *m* *M* ATP, Sigma-Aldrich-A7699) to a final 15 *μ* *l* volume. The thermocycler program was set to: 3 cycles of 10 min at 40 °C and other 10 min at 16 °C. These cycles were followed by 10 min at 50 °C, 20 min at at 80 °C, and the final temperature was set to 16 °C. *E. coli* competent cells (strain DH5 *α*, Life Technology 18263-012) transformed with our plasmids (30-s heatshock at 42 °C) were grown overnight at 37 °C either in LB broth or plates (Bacto-tryptone 10%, Yeast extract 5%, NaCl 10%, Agar 15% for the plates) supplied with the necessary antibiotic (ampicillin or spectinomycin). Plates were spread with 100*μ* *l*100 *m* *M* IPTG (Merck) and 100*μ* *l*20 *m* *g*/*m* *l* X-gal for blue/white screening. Plasmid extraction from bacterial cells was carried out with standard methods [37]. All the plasmids were further sequenced (Sanger method) to check the correctness of the newly assembled synthetic constructs.

### Yeast strain construction

Our new, synthetic plasmids were integrated into the genome of the yeast *S. cerevisiae* strain FY1679-08A (MATa; ura3-52; leu2 *Δ*1; trp1 *Δ*63; his3 *Δ*200; GAL2), Euroscarf (Johann Wolfgang Goethe University, Frankfurt, Germany). Genomic integration was carried out as described in [38]. About 5 *μ* *g* of plasmidic DNA were linearized either along the URA3 marker with the restriction enzyme StuI (NEB-R0187S) or along the LEU2 marker with the restriction enzyme BstXI (NEB-R0113S). Transformed cells were grown on plates containing synthetic selective medium (SD-URA or SD-LEU; 2% glucose, 2% agar) for about 36 h at 30 °C.

### Flow cytometry

Yeast cells were grown overnight in synthetic complete medium (SDC) at 30 °C. They were diluted, in the morning, approximately 1:100 and let them grow (in synthetic medium again) up to five more hours such that their *O* *D*_600_ was always between 0.2 and 2.0 (exponential phase). Fluorescence measurements were performed with a BD FACScalibur machine (488 *n* *m* laser, 530/30 filter). The FACS machine set-up was reproduced at each experiment by using fluorescent beads (AlignFlow, Life Technologies-A16500). We placed their peak (mean value) as close as possible to 400 AU. The measurement was repeated at the end of each experiment to assure that the machine conditions did not change considerably over the whole experiment. We considered as reliable only the measurements where the relative difference between the initial and the final value of the peaks of the beads was lower than 5%. Data were analyzed with the flowcore R-Bioconductor package [39]. Statistically significant difference between two fluorescence levels was estimated via two-sided Welch’s t-test (*p*-value <0.05). Fluorescence levels were estimated as the mean values of at least three independent experiments (i.e. carried out in different days–each time 30000 samples were recorded). Standard deviations were calculated on these mean values. The error on a relative fluorescence value (ratio) was finally computed via the error propagation formula. Box plots and histograms of representative experiments are provided in the Additional file 1.

## Additional file

Additional file 1 Supplementary Material. (PDF 494 kb)
