# Supplementary material for: Can terminators be used as insulators into yeast synthetic gene circuits?
Source: J Biol Eng. 2016 Dec 16;10:19. doi: 10.1186/s13036-016-0040-5 (PMC5162094; doi:10.1186/s13036-016-0040-5)
Supplement: Supplementary file 1 — Supplementary Material. (PDF 494 kb) [file 13036_2016_40_MOESM1_ESM.pdf]

Can terminators be used as insulators into yeast synthetic gene circuits?
Supplementary Material

Wenjiang Song[1], Jing Li[1], Qiang Liang[1], and Mario Andrea Marchisio[1,*]

[1]School of Life Science and Technology, Harbin Institute of Technology,

2 Yikuang Street, Nan Gang District, 150080 Harbin, P. R. China

[*]corresponding author

email addresses: WS, 1176784021@qq.com; JL, 920138462@qq.com; QL, 804257847@qq.com;

MAM: marchisio@hit.edu.cn, mamarchisio@yahoo.com

Results and Discussion

Building new synthetic promoters by using terminator sequences

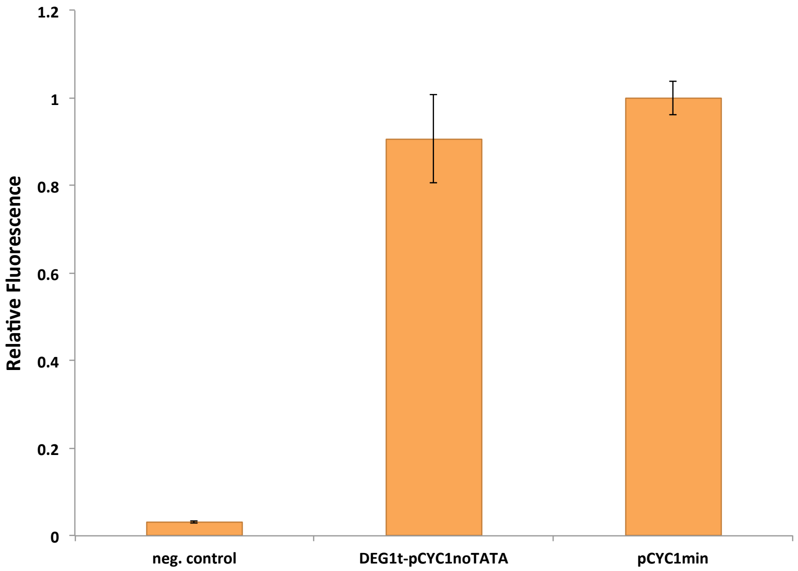

Figure S1: Comparison between the synthetic DEG1t-pCYC1noTATA and the minimal CYC1 promoter. Fluorescence levels are normalized with respect to the one of pCYC1min. DEG1t-pCYC1noTATA and pCYC1min seem to have the same strength since no statistically significant difference between their fluorescence levels was detected (two-sided Welch's t-test, p-value = 0.94). Compared to pCYC1min, DEG1t-pCYC1noTATA has a stronger TATA box at a slightly longer distance (7 more nucleotides) from the TSS.

Insulating the strong GPD promoter

Table S1: Distance between the terminator TATA-box motifs and the pGPD strong bipartite UAS (nt stands for nucleotides).

Promoter	TATA box	Kind	Distance from pGPD UAS (nt)
DEG1t-pGPD	TATAAA	Strong	205
Tsynth8-pGPD	TATAAA	Strong	209
genCYC1t-pGPD	TATTTA	Weak	264
	TATTAA	Weak	236
	TATTTA	Weak	224
shortADH1t-pGPD	TATAAAA	Strong	312
	TTTAAA	Weak	287
	TTTAAA	Weak	268

Modeling

As we have shown in the main text, the insulation of GPD promoter with the DEG1 terminator provokes a remarkable decrease in gene expression. In our view, this is due to the fact that DEG1t yeast *S. cerevisiae* terminator contains a strong efficiency element that resembles and works as a strong TATA box i.e. it recruits RNA polymerase II molecules. This was proved with the insulation of the promoter we termed pCYC1noTATA. RNA polymerase II molecules, by binding the insulator efficiency element, interfere with promoter transcriptional activity. There are two possibilities: 1) the DEG1t efficiency element and the pGPD TATA box bind RNA polymerase II independently i.e. DEG1t efficiency element contributes only to a reduction in the number of RNA polymerase II molecules available in the nucleus (*independent binding*); 2) the efficiency element, by binding RNA polymerase II, sequesters the promoter and prevents other RNA polymerase II molecules from binding the TATA box (*promoter sequestration*). It should be noted that the binding of the activator GRF1 to the pGPD UAS is not taken, here, explicitly into account. However, its contribution to the transcription process, namely the recruitment of RNA polymerase II molecules to the pGPD TATA-box, is present in the model. Our analysis aims only at showing which one of the two possibilities described above can represent properly our experimental results i.e. the reduction in fluorescence expression due to pGPD insulation by DEG1t. To this aim, we do not need a complete mechanistic description of transcription initiation that takes into account all the factors that help RNA polymerase II bind the DNA and other mechanisms such as DNA bending.

Independent binding

Independent binding of RNA polymerase II to DEG1t terminator efficiency element and pGPD TATA box can be modeled as

$$\begin{aligned} Pol + P &\overset{(k_1,k_{-1})}{\rightleftharpoons} P^* \\ P^* &\xrightarrow{k_2} Pol + P + F \\ F &\xrightarrow{k_d} \\ Pol + E &\overset{(\alpha,\beta)}{\rightleftharpoons} E^*\,, \end{aligned} \tag{1}$$

where Pol represents RNA polymerase II molecules available in the nucleus, P is the promoter in its inactive configuration (RNA polymerase II is not bound to the TATA-box), P^* is the active promoter (RNA polymerase is bound to the TATA box), F is the fluorescence protein, E represents the terminator efficiency element free of any RNA polymerase II molecules, and E^* is the terminator efficiency element bound to RNA polymerase II. Rate -constant symbols and units are explained in Table S2. As stated above, the role of the efficiency element in Eqs (1) is to lower the number of RNA polymerase II molecules that can contribute to fluorescence expression. For the sake of simplicity, in Eqs (1) translation is treated as a single step event and cell compartmentalization is neglected.

Rate constant	Reaction	Units
k_1	RNA polymerase II-promoter association	$M^{-1}s^{-1}$
k_{-1}	RNA polymerase II-promoter dissociation	s^{-1}
k_2	transcription initiation	s^{-1}
k_d	GFP decay	s^{-1}
α	RNA polymerase II-insulator association	$M^{-1}s^{-1}$
β	RNA polymerase II-insulator dissociation	s^{-1}

Table S2: Rate constants used in Eqs (1).

We assume that the cell volume corresponds to the yeast nuclear volume ($V = 2.9\,10^{-15}\,l$ [1]). We integrated a single copy of each construct into the yeast genome. Hence, we shall require

that $P_T = P + P^* = 1$ i.e. the total number of promoter molecules (P_T) is equal to 1. Since the green fluorescence protein (GFP) is very stable, we can take as a reasonable approximation for its half-life the doubling time of yeast cells in a synthetic medium, i.e. 140 minutes [2]. This corresponds to $k_d = 8.25\,10^{-5}\,s^{-1}$. In the lab, we did not measure GFP concentration but only fluorescence levels. To our knowledge, there is not precise way to converts a fluorescence level (which is strongly affected by the machine type and setup) to a protein number. Since pGPD is a strong promoter, we think that is it reasonable to consider that, at steady state, at least 100 molecules of fluorescent proteins are present in the cell [3]. In the following analysis we will use two possible GFP steady-state amounts: 100 (A) and 1000 (B) molecules. We will see that the qualitative result of our model (i.e. whether independent binding or promoter sequestration takes place) is independent of GPF concentration at steady state (provided that is as high as we assume). Values for k_1, k_{-1}, k_2, and Pol_T (i.e. the total number of RNA polymerase II molecules available in the cell to bind either P or E) are found by running an optimization procedure on the first three reactions in Eqs (1). They represent a model for the dynamics of GFP produced by the constitutive GPD promoter. We run the Simulated Annealing (SA) algorithm implemented in COPASI [4] (see TableS5 for our setting of calculation parameters) and set the lower and upper bound of each kinetic parameter according to the values in [1] (see Table S3). For each of the two chosen GFP steady states (100 and 1000 molecules), we repeated the optimization procedure three times (I, II, III) by varying each time the upper and lower bound on Pol_T (see Table S4 [1]). The objective function was set either to $(F_{ss} - 100)^2 = 0$ (case A) or $(F_{ss} - 1000)^2 = 0$ (case B), where F_{ss} is the number of GFP particles at steady state.

Rate constant	Lower bound	Upper bound	Units
k_1	10^4	10^7	$M^{-1}s^{-1}$
k_{-1}	10^{-3}	10	s^{-1}
k_2	0.1	2	s^{-1}
α	10^4	10^9	$M^{-1}s^{-1}$
β	10^{-4}	10	s^{-1}

Table S3: Upper and lower bound on the kinetic parameter values used in our models.

Label	Lower bound	Upper bound
I	80	100
II	1600	2000
III	4000	5000

Table S4: Our choices of Pol_T upper and lower bound.

Parameter	Value
Starting Temperature	1
Cooling Factor	0.85
Tolerance	10^{-6}
Random Number Generator	1
Seed	0

Table S5: Simulated Annealing parameter setting.

After finding, on the whole, six possible sets of parameters values for k_1, k_{-1}, k_2, and Pol_T (see Table S6), we considered the complete model in Eqs (1) and looked for values of α and β that could reproduce the wet-lab result of the DEG1t-pPGPD system i.e. a fluorescence level equal to the 77 % of the non-insulated one. To this aim, we made use of Simulated Annealing again. Upper and lower bounds for both α and β, reported in Table S3, are similar to the one for k_1 and k_{-1}, respectively. However, we set a higher upper bound on α and a smaller lower bond on

Parameter set	F_ss	k_1	k_{-1}	k_2	Pol_T
A.I	100	$1.56\,10^5$	0.026	1.71	93.99
A.II	100	$1.21\,10^4$	0.084	0.19	1787.29
A.III	100	$1.22\,10^4$	0.994	0.41	4116.89
B.I	1000	$1.52\,10^6$	0.007	1.73	99.80
B.II	1000	$7.86\,10^4$	0.031	1.37	1994.56
B.III	1000	$3.78\,10^4$	0.027	1.96	4037.8

Table S6: Parameter values–obtained via Simulated Annealing–for GFP constituive expression under GPD promoter (first three reactions in Eqs (1)).

β to allow a stronger binding between RNA polymerase II and the efficiency element. On each of our six parameter sets SA failed to find values for α and β such that the objective function was satisfied (see Table S7). Therefore, we had to conclude that the *independent binding* model could not explain our experimental data.

Parameter set	α	β	F_{ss}	Target F_{ss}
A.I	$1.00\,10^9$	0.0001	99.11	77
A.II	$9.97\,10^8$	0.0001	99.97	77
A.III	$9.97\,10^8$	0.0001	99.97	77
B.I	$1.00\,10^9$	0.0001	989.87	770
B.II	$1.00\,10^9$	0.0001	999.53	770
B.III	$9.99\,10^8$	0.0001	999.76	770

Table S7: Values for α and β in Eqs (1) computed by SA and the corresponding GFP particle number at steady state (F_{ss}–calculated by COPASI by solving the ODE system in Eqs(3)). "Target F_{ss}" is the result the model should achieve after the optimization procedure. However, every model returns an F_{ss} that is about 1.3 folds higher than the target one.

Promoter sequestration

In the promoter sequestration model, RNA polymerase II, by binding the efficiency element, prevents other RNA polymerase II molecules from binding the TATA box and start F synthesis. Reactions and species become

$$\begin{aligned} Pol + EP &\overset{(k_1,k_{-1})}{\rightleftharpoons} EP^* \\ EP^* &\overset{k_2}{\longrightarrow} Pol + EP + F \\ F &\overset{k_d}{\longrightarrow} \\ Pol + EP &\overset{(\alpha,\beta)}{\rightleftharpoons} E^*P\,. \end{aligned} \quad (2)$$

According to Eqs (2), efficiency element (E) and TATA box (P) belong to a unique species (EP) where RNA polymerase II can bind either site: EP^* is the active promoter configuration, i.e. RNA polymerase II has bound the TATA box and can proceed with gene expression, whereas E^*P is the sequestered promoter, where RNA polymerase II has bound the insulator and prevents promoter activation. As we did in the previous model, we run SA on the six sets of values for k_1, k_{-1}, k_2, and Pol_T in Table S6 looking for values of α and β that could reproduce our wet-lab data. Upper and lower bound for α and β are the same as in Table S3. In the promoter sequestration framework, SA was always able to find values for α and β such that F_{ss} was equal to either 77 or 770 molecules (see Table S8).
On the whole, although the models in Eqs (1,2) are based on a very simplified picture of transcription initiation and protein synthesis, they point out clearly that, in order to explain our experimental results, we have to assume that a terminator, when used as an insulator, prevents

Parameter set	α	β	F_{ss}	Target F_{ss}
A.I	$8.83\,10^5$	0.159	77	77
A.II	$2.92\,10^4$	0.096	77	77
A.III	$3.00\,10^4$	0.232	77	77
B.I	$1.46\,10^5$	0.027	770	770
B.II	$2.21\,10^5$	0.794	770	770
B.III	$1.06\,10^5$	0.788	770	770

Table S8: The values of α and β–in Eqs (2)–computed by SA satisfy the request that F_{ss} (a solution of the ODE system in Eqs(4) is equal to "Target F_{ss} ".

a full promoter activation. RNA polymerase II, by binding the insulator efficiency element, sequesters the promoter and limits the binding of other RNA polymerase II molecules to the TATA box. This result does not require a precise knowledge of the amount of RNA polymerase II in the cell and the number of green fluorescent proteins at steady state. The parameters involved in our models have been tuned to reproduce the case of the insulation of the strong GPD promoter by means of the DEG1t terminator. However, this theoretical representation can be applied to different insulated promoters too.

ODE systems

The name of each species in the following ODE systems indicates species concentration.

Independent binding

$$\begin{aligned}
\frac{dPol}{dt} &= -k_1 Pol\,P + (k_{-1}+k_2)P^* - \alpha Pol\,E + \beta E^* \\
\frac{dP}{dt} &= -k_1 Pol\,P + (k_{-1}+k_2)P^* \\
\frac{dP^*}{dt} &= k_1 Pol\,P - (k_{-1}+k_2)P^* \\
\frac{dF}{dt} &= k_2 P^* - k_d F \\
\frac{dE}{dt} &= -\alpha Pol\,E + \beta E^* \\
\frac{dE^*}{dt} &= \alpha Pol\,E - \beta E^* \qquad (3)
\end{aligned}$$

Promoter sequestration

$$\begin{aligned}
\frac{dPol}{dt} &= -k_1 Pol\,EP + (k_{-1}+k_2)EP^* - \alpha Pol\,EP + \beta E^*P \\
\frac{dEP}{dt} &= -k_1 Pol\,EP + (k_{-1}+k_2)EP^* \\
\frac{dEP^*}{dt} &= k_1 Pol\,EP - (k_{-1}+k_2)EP^* \\
\frac{dF}{dt} &= k_2 EP^* - k_d F \\
\frac{dEP}{dt} &= -\alpha Pol\,EP + \beta E^*P \\
\frac{dE^*P}{dt} &= \alpha Pol\,EP - \beta E^*P \qquad (4)
\end{aligned}$$

Insulating different promoters

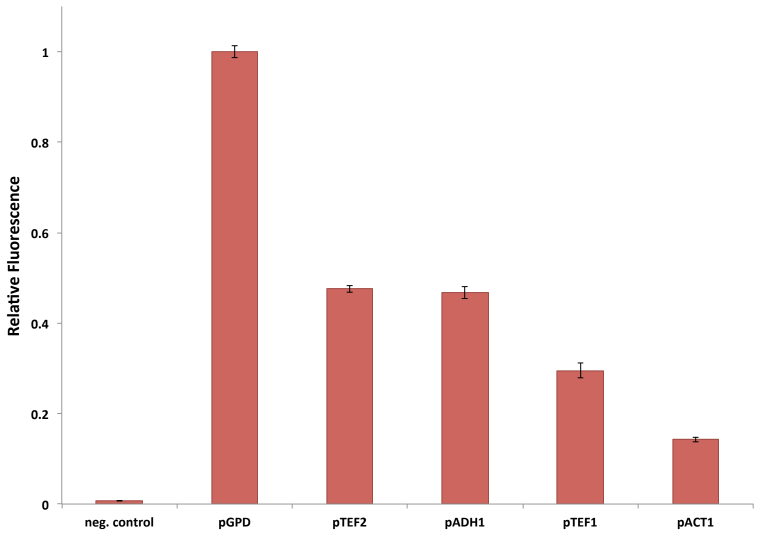

Figure S2: Yeast constitutive promoter strength. Comparison of the fluorescence level associated with the five yeast constitutive promoters used in this work. All fluorescence levels are normalized with respect to the pGPD one.

Placing an insulator between two transcription units

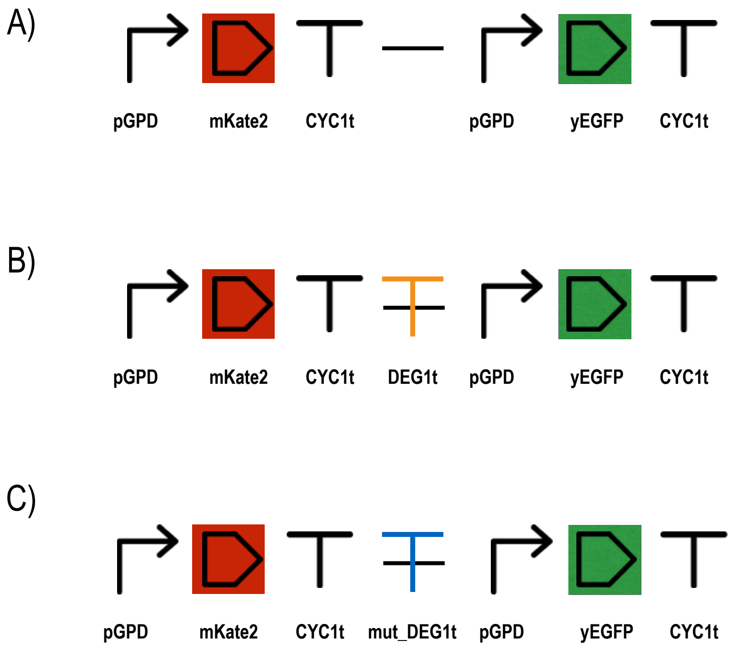

Figure S3: Two-transcription-unit systems. Two adjacent transcription units are assembled inside a vector. Both contain pGPD and CYC1t. The upstream transcription unit expresses red fluorescent protein, whereas the downstream transcription unit expresses the green one. A) No insulator is placed between the two transcription units. B) DEG1t lies between the two transcription units. C) mut_DEG1t replaces DEG1ts between the two transcription units.

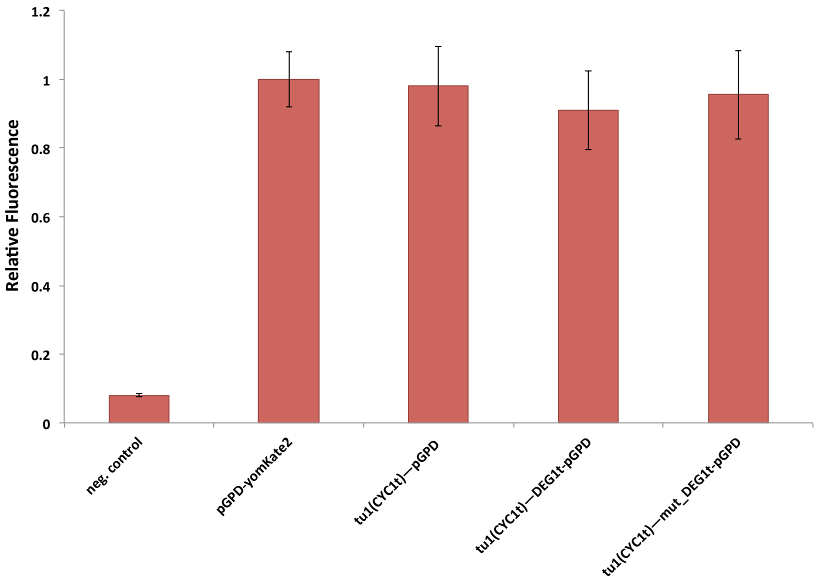

Figure S4: Red fluorescent protein expression by two-transcription-unit systems. yomKate2 expression seems not to resent of the presence of a downstream transcription unit, not matter if insulated or not. Each two-transcription-unit construct is labeled as tu1(CYC1) (i.e. the upstream transcription unit that encodes for yomKate2 and ends with CYC1t) and the name of the promoter of the downstream transcription unit. The positive control is the strain that produces yomKate2 only (pGPD-yomKate2). Fluorescence levels are normalized with respect to the one expressed by pGPD in a single transcription unit.

Methods

Assembly techniques: GFP expression and difference in plasmid sequences

As shown in Table S9, most of our plasmids were constructed with the isothermal assembly technique (ITA) and few of them with the MoClo method. The 1-level acceptor vector ypL1F-1_406 was built on pRSII406 as explained in the main text. Moreover, a BsaI site was removed from the ampicillin resistance sequence and another BsaI site together with a BpiI site were removed from the URA3 marker sequence. In the plasmids assembled with the MoClo method, a transcription unit is flanked by two BpiI sites, whereas in the plasmids assembled through the isothermal assembly technique transcription units are placed between KpnI and SacI sites. Each transcription unit assembled with the MoClo method has four more bases (GCTT) between yEGFP and CYC1t. Finally, the plasmids assembled with the MoClo method contains yEGFPgg where a BsaI site was removed via a silent mutation. In order to check if these differences had an effect on GFP expression, we assembled a transcription unit carrying DEG1t-pCYC1noTATA both into pMM146 (ITA and yEGFP) and pMM195 (MoClo and yEGPgg). These two plasmids were integrated into the yeast strain byMM2 giving raise to byMM67 and byMM88, respectively. Their fluorescence levels, shown in Figure S5, do not have any statistically significant difference. Therefore, our results are independent of the plasmid assembly techniques.

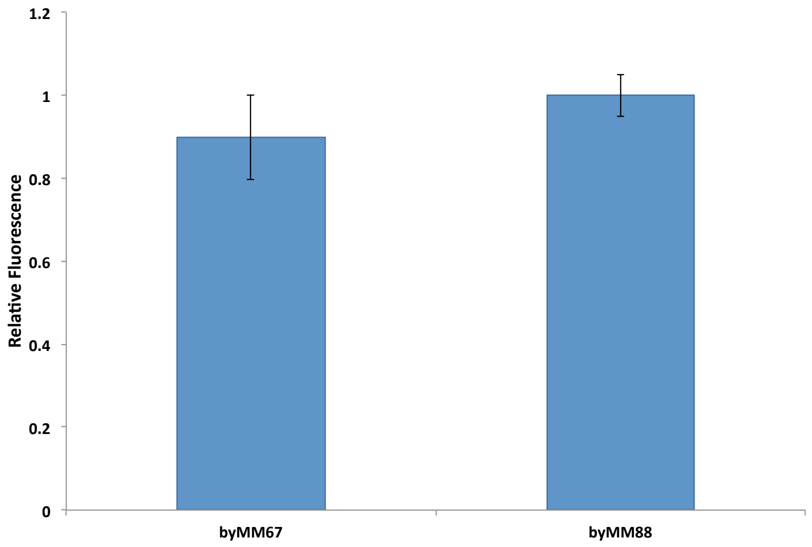

Figure S5: Comparison of fluorescence levels from plasmids assembled with different techniques. The transcription unit into pMM195 (byMM88) differs from the one into pMM146 (byMM67) for a point mutation along the yEGFP sequence and four more bases before CYC1t. Few more differences in the two plasmids are due to the adaptation of pRSII406 to the MoClo procedure. All these modifications do not to have any particular influence on protein expression since we did not find any statistically significant difference between the fluorescence levels of the two strains (two-sided Welch's t-test, p-value = 0.28). Fluorescence levels are normalized with respect to byMM88 one.

Plasmids used in this work

Plasmid name	Construct	Assembly method
pMM53	pRSII406-pGPD-yEGFP-CYC1t	ITA
pMM54	pRSII406-DEG1t-pGDP-yEGFP-CYC1t	ITA
pMM61	pRSII406-pCYC1min-yEGFP-CYC1t	ITA
pMM72	pRSII406-genCYC1t-pGPD-yEGFP-CYC1t	ITA
pMM73	pRSII406-Tsynth8-pGPD-yEGFP-CYC1t	ITA
pMM81	pRSII406-shortADH1t-pGPD-yEGFP-CYC1t	ITA
pMM90	pRSII406-pTEF1-yEGFP-CYC1t	ITA
pMM109	pRSII406-pCYC1noTATA-yEGFP-CYC1t	ITA
pMM123	pRSII406-pACT1-yEGFP-CYC1t	ITA
pMM124	pRSII405-pGPD-yomKate2-CYC1t	ITA
pMM131	pRSII406-pTEF2-yEGFP-CYC1t	ITA
pMM132	pRSII406-Tsynth8-pCYC1noTATA-yEGFP-CYC1t	ITA
pMM137	ypL1F-1_406 (yeast 1-level acceptor; URA3)	ITA
pMM144	pRSII406-shortADH1t-pCYC1noTATA-yEGFP-CYC1t	ITA
pMM146	pRSII406-DEG1t-pCYC1noTATA-yEGFP-CYC1t	ITA
pMM188	ypL1F-1_406-genCYC1t-pCYC1noTATA-yEGFPgg-CYC1t	MoClo
pMM194	ypL1F-1_406-Tsynth8-s100-pCYC1noTATA-yEGFPgg-CYC1t	MoClo
pMM195	ypL1F-1_406-DEG1t-pCYC1noTATA-yEGFPgg-CYC1t	MoClo
pMM284	pRSII406-mut_Tsynth8-pCYC1noTATA-yEGFP-CYC1t	ITA
pMM285	pRSII406-mut_DEG1t-pCYC1noTATA-yEGFP-CYC1t	ITA
pMM292	pRSII406-mut_pGPD-yEGFPgg-CYC1t	ITA
pMM294	pRSII406-mut_DEG1t-PGPD-yEGFP-CYC1t	ITA
pMM326	ypL1F-1_406-pGPD-yomKate2-CYC1t-pGPD-yEGFPgg-CYC1t	MoClo
pMM340	ypL1F-1_406-pGPD-yomKate2-CYC1t-DEG1t-pGPD-yEGFPgg-CYC1t	MoClo
pMM342	pRSII406-DEG1t-mut_PGPD-yEGFPgg-CYC1t	ITA
pMM343	pRSII406-mut_DEG1t-mut_PGPD-yEGFPgg-CYC1t	ITA
pMM344	ypL1F-1_406-pGPD-yomKate2-CYC1t-mut_DEG1t-pGPD-yEGFPgg-CYC1t	MoClo
pMM345	pRSII406-DEG1t-pTEF2-yEGFP-CYC1t	ITA
pMM346	pRSII406-DEG1t-pTEF1-yEGFP-CYC1t	ITA
pMM347	pRSII406-DEG1t-pACT1-yEGFP-CYC1t	ITA
pMM354	pRSII406-pADH1-yEGFPgg-CYC1t	ITA
pMM376	pRSII406-DEG1t-pADH1-yEGFPgg-CYC1t	ITA

Table S9: List of the plasmids constructed in this work.

Yeast strain used in this work

Strain name	Genotype
byMM2	FY1679-08A
byMM8	byMM2 pMM53::URA3
byMM10	byMM2 pMM54::URA3
byMM15	byMM2 pMM61::URA3
byMM19	byMM2 pMM72::URA3
byMM24	byMM2 pMM73::URA3
byMM33	byMM2 pMM81::URA3
byMM38	byMM2 pMM90::URA
byMM54	byMM2 pMM109::URA3
byMM57	byMM2 pMM123::URA3
byMM60	byMM2 pMM131::URA3
byMM61	byMM2 pMM132::URA3
byMM66	byMM2 pMM144::URA3
byMM67	byMM2 pMM146::URA3
byMM88	byMM2 pMM195::URA3
byMM89	byMM2 pMM188::URA3
byMM90	byMM2 pMM194::URA3
byMM119	byMM2 pMM124::LEU2
byMM122	byMM2 pMM284::URA3
byMM123	byMM2 pMM285::URA3
byMM147	byMM2 pMM294::URA3
byMM148	byMM2 pMM344::URA3
byMM149	byMM2 pMM292::URA3
byMM150	byMM2 pMM342::URA3
byMM151	byMM2 pMM343::URA3
byMM162	byMM2 pMM326::URA3
byMM163	byMM2 pMM340::URA3
byMM179	byMM2 pMM346::URA3
byMM184	byMM2 pMM347::URA3
byMM193	byMM2 pMM345::URA3
byMM194	byMM2 pMM354::URA3
byMM196	byMM2 pMM376::URA3

Table S10: List of the yeast strains constructed in this work. Plasmids are described in Table S9.

Data analysis: box plots and histograms.

A

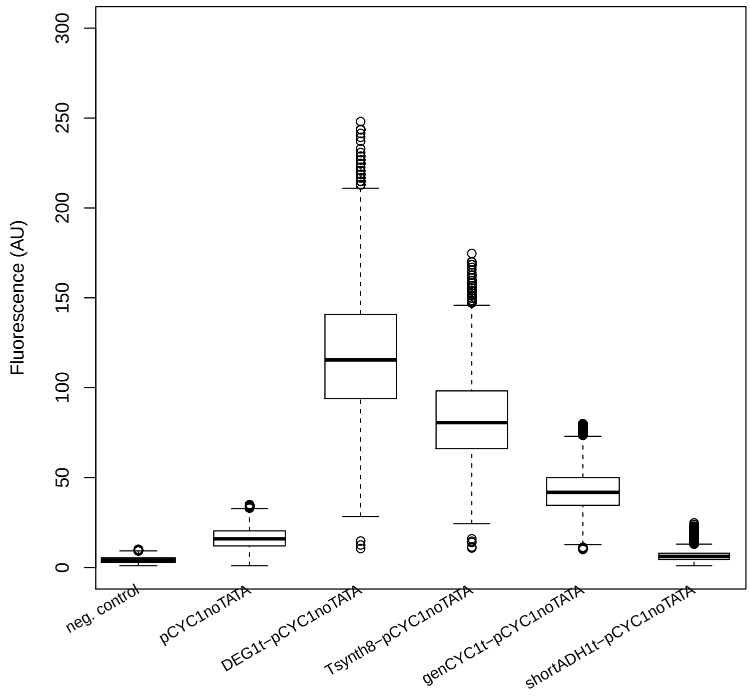

B

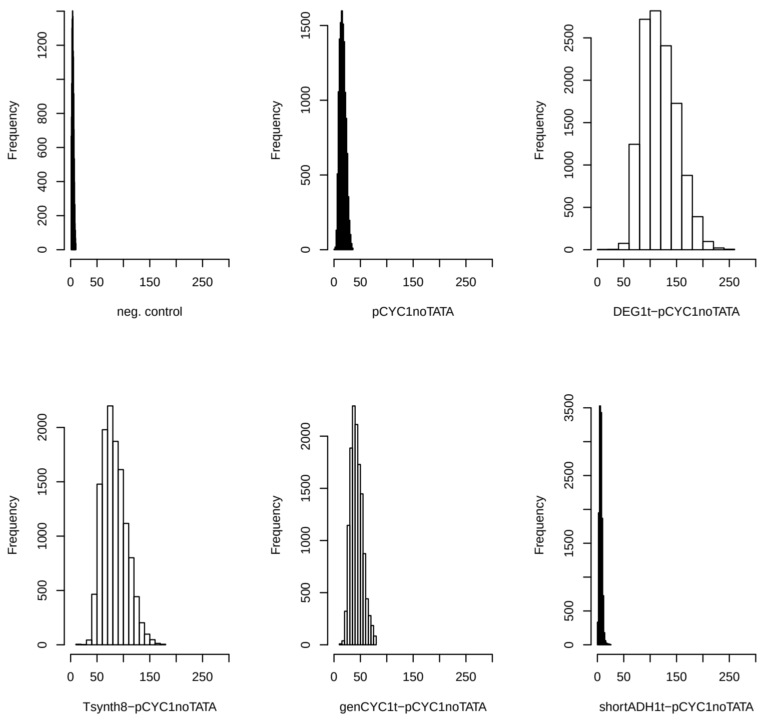

Figure S6: Fluorescence levels for the characterization of synthetic promoters made of a terminator placed in front of pCYC1noTATA (see Figure 3 in the main text). A) Box plots. B) Histograms.

A

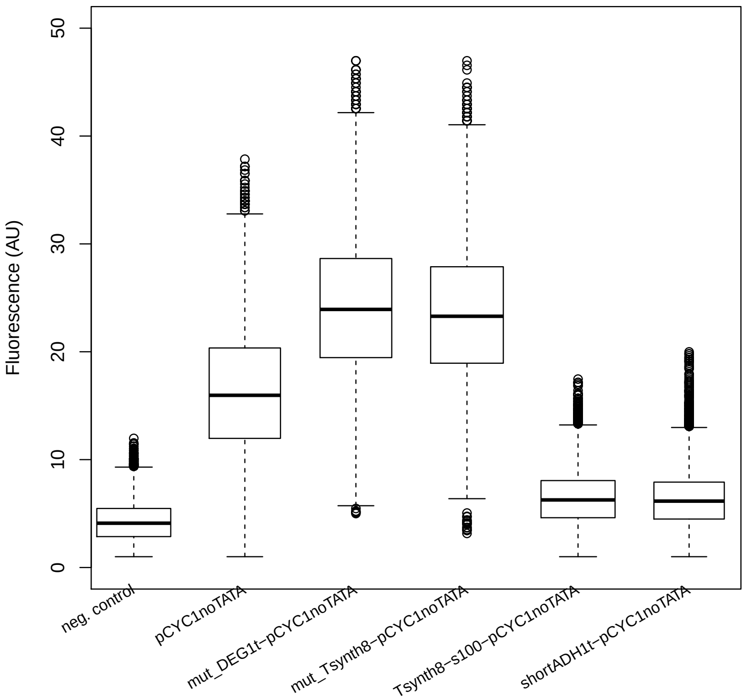

B

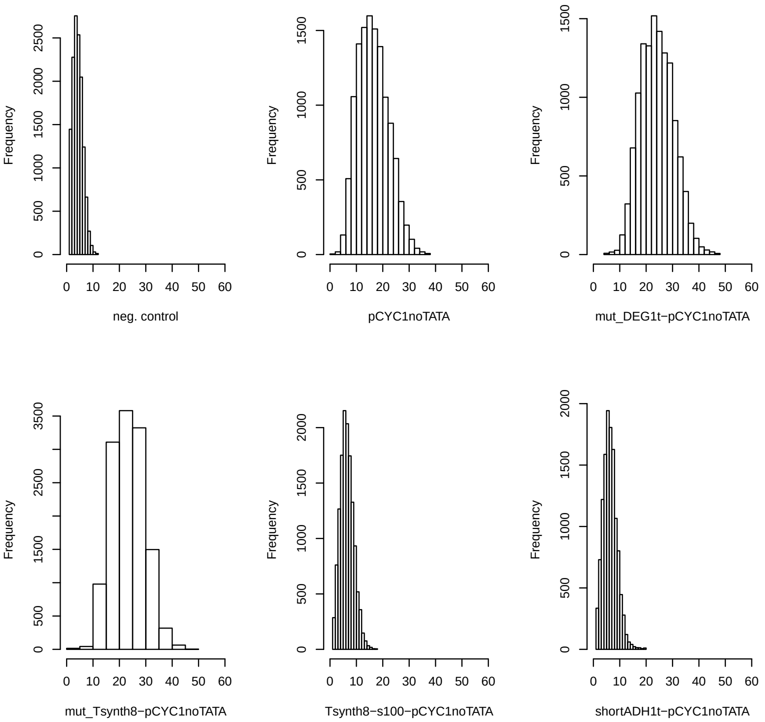

Figure S7: Mutational study on DEG1t-pCYCnoTATA and Tsynth8-pCYC1noTATA (see Figure 4 in the main text). A) Box plots. B) Histograms.

A

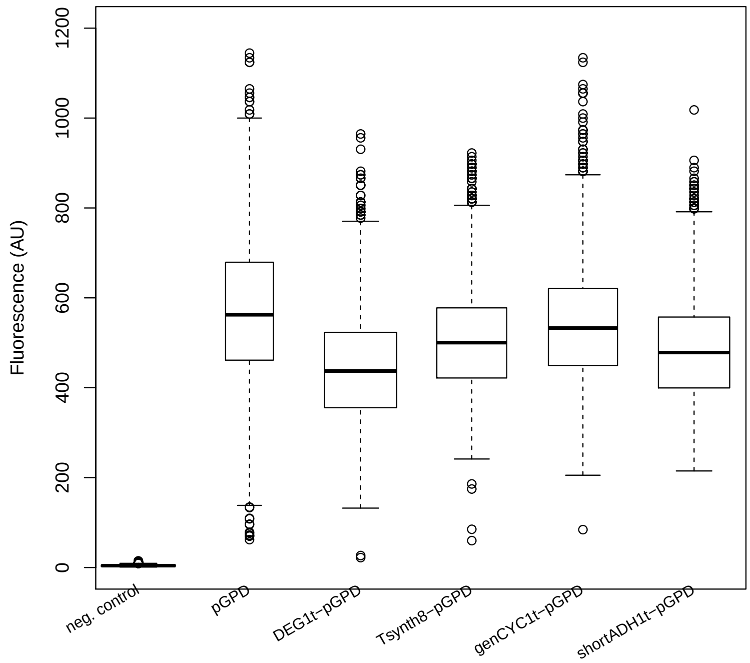

B

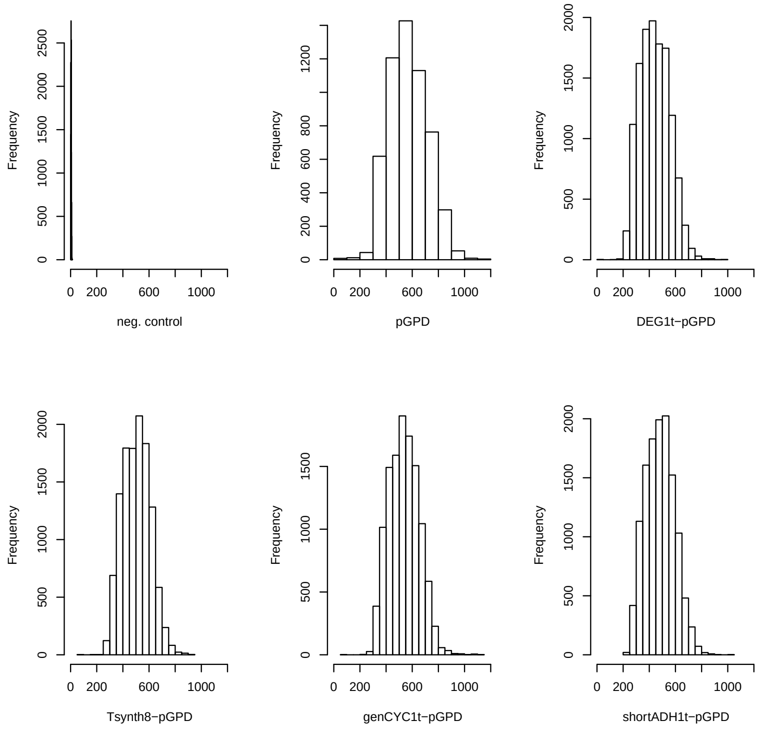

Figure S8: Fluorescence levels expressed by synthetic promoters made of different terminators preceding the strong GPD promoter (see Figure 7 in the main text). A) Box plots. B) Histograms.

A

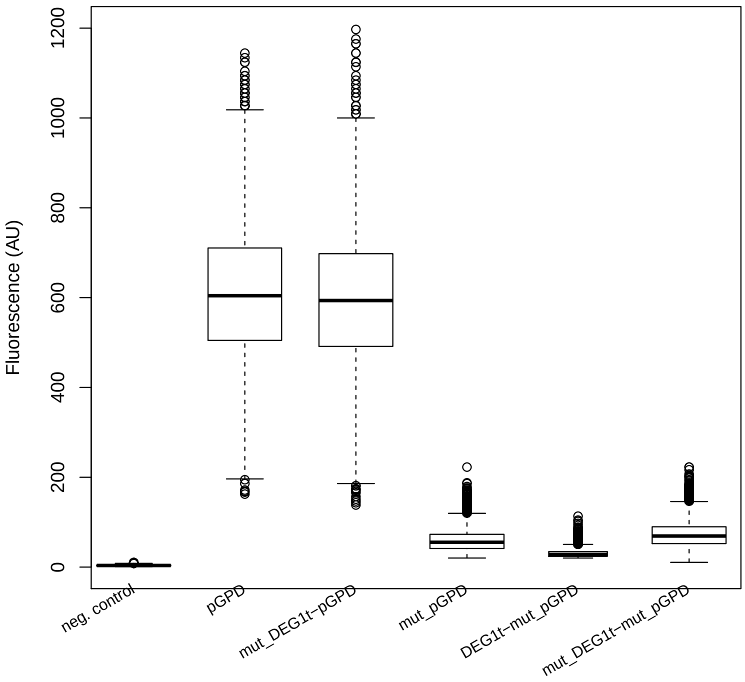

B

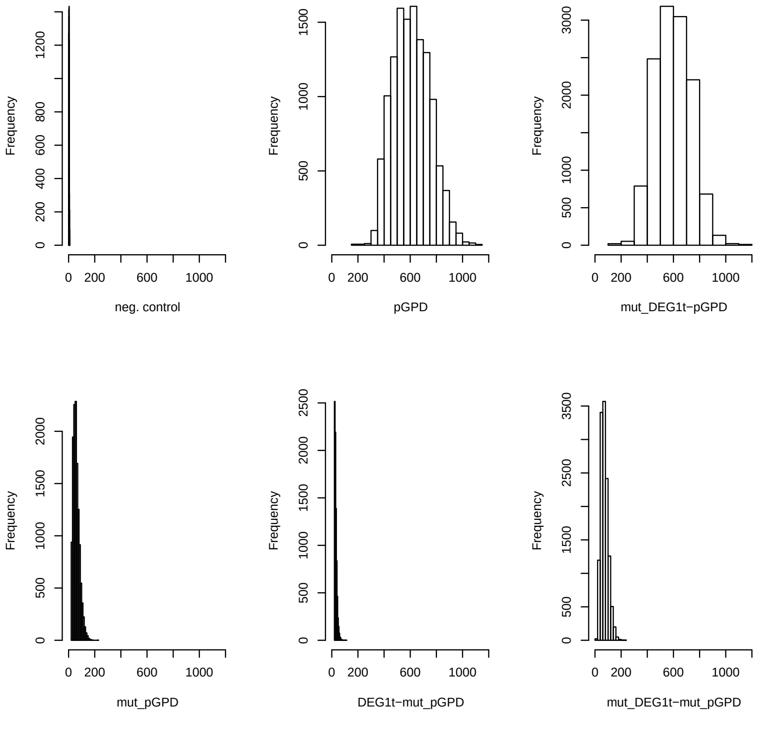

Figure S9: Mutational study on DEG1t-pGPD and DEG1t-mut_pGPD. (see Figure 8 in the main text). A) Box plots. B) Histograms.

A

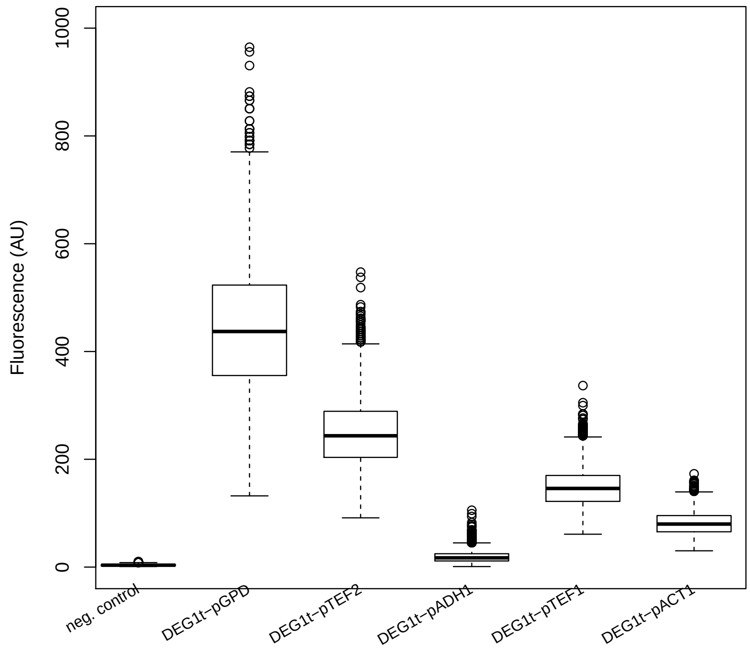

B

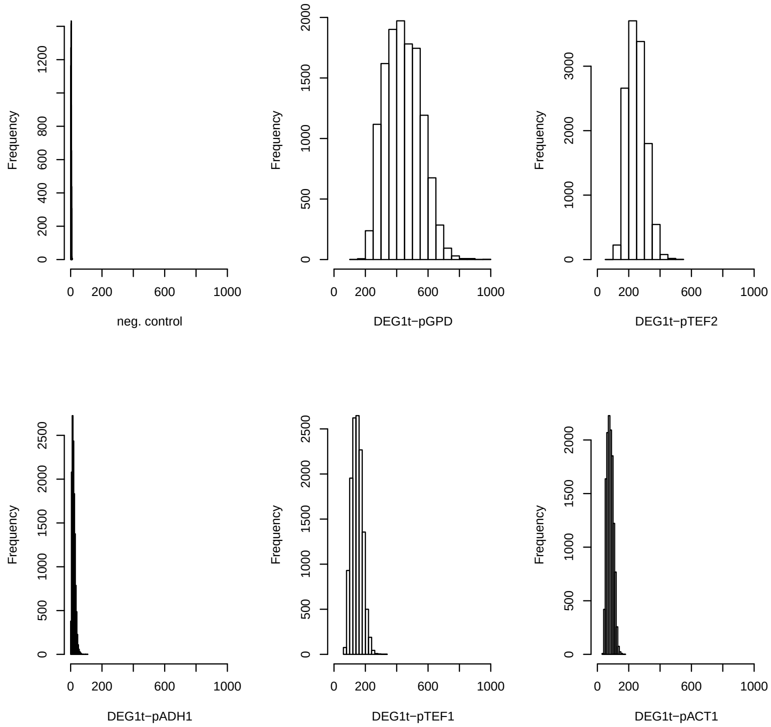

Figure S10: Effects of DEG1t as an insulator of five different yeast constitutive promoters (see Figure 9 in the main text). A) Box plots. B) Histograms.

A

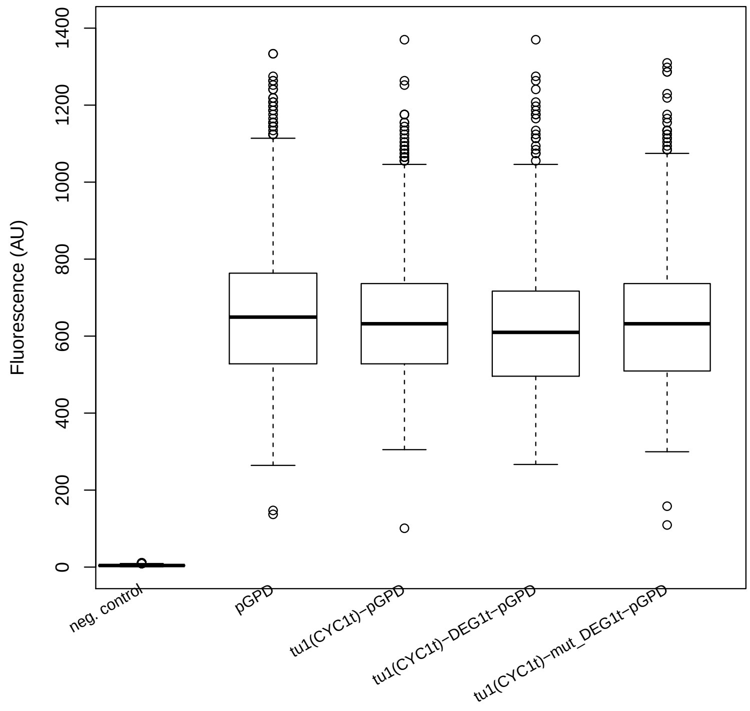

B

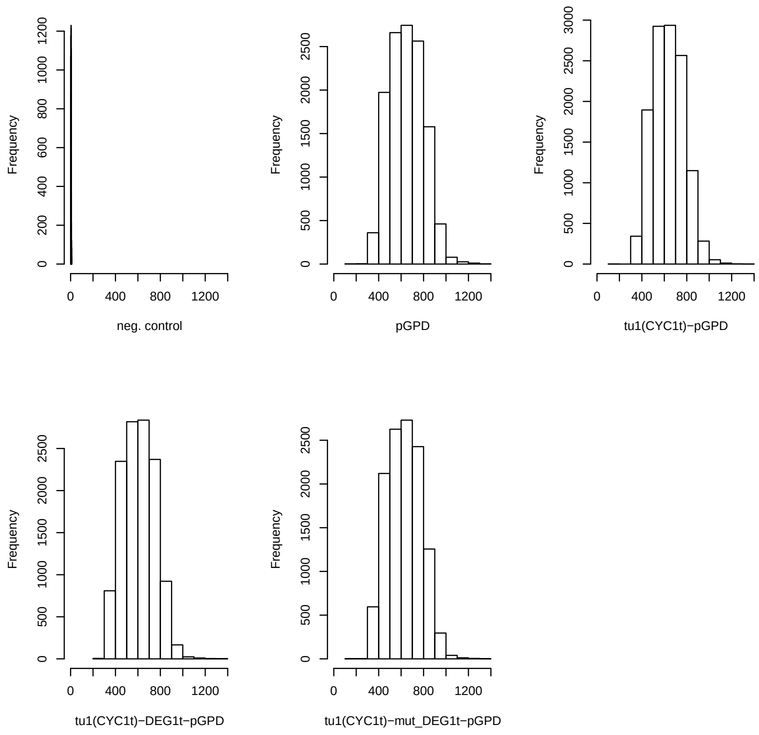

Figure S11: Expression of green fluorescence by two-transcription-unit systems (see Figure 10 in the main text). A) Box plots. B) Histograms.

A

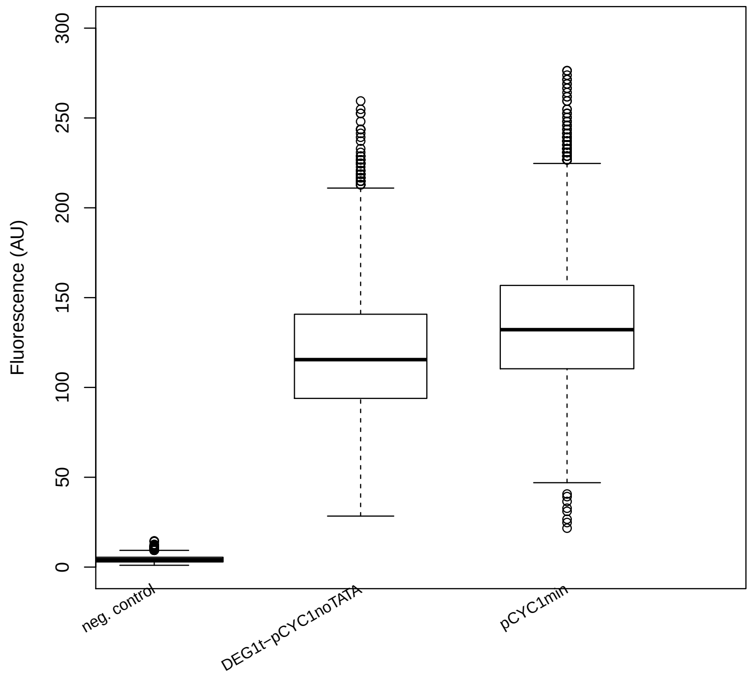

B

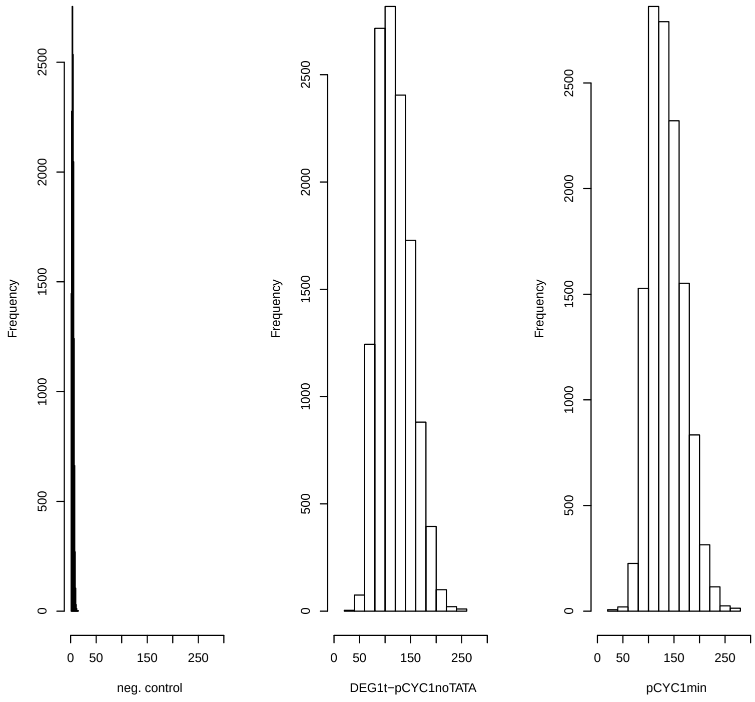

Figure S12: Comparison of the synthetic DEG1t-pCYC1noTATA with the minimal CYC1 promoter (see Figure S1 above). A) Box plots. B) Histograms.

A

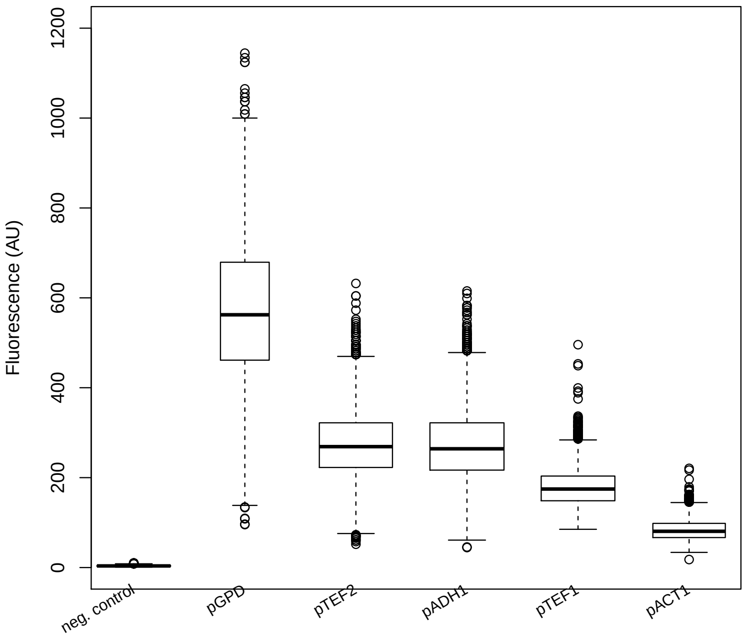

B

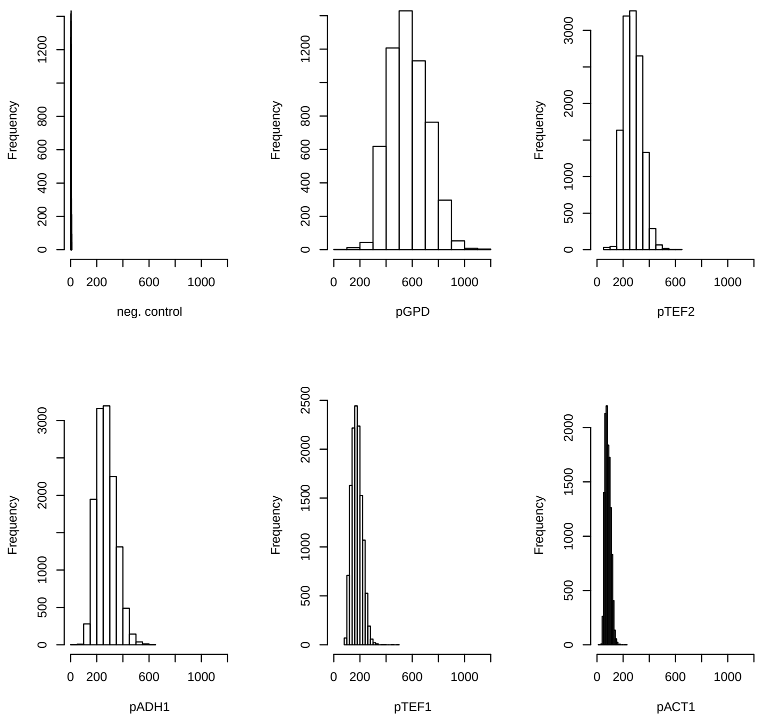

Figure S13: Yeast constitutive promoter strength (see Figure S2 above). A) Box plots. B) Histograms.

A

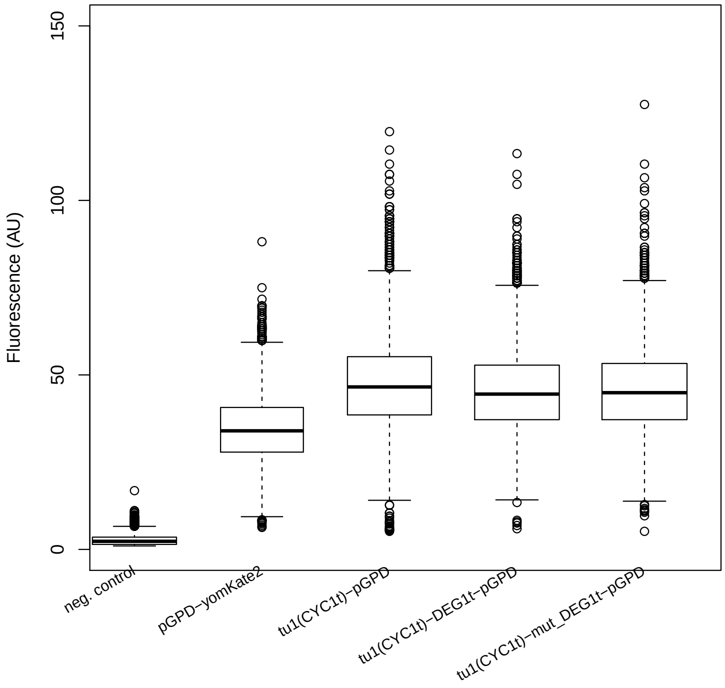

B

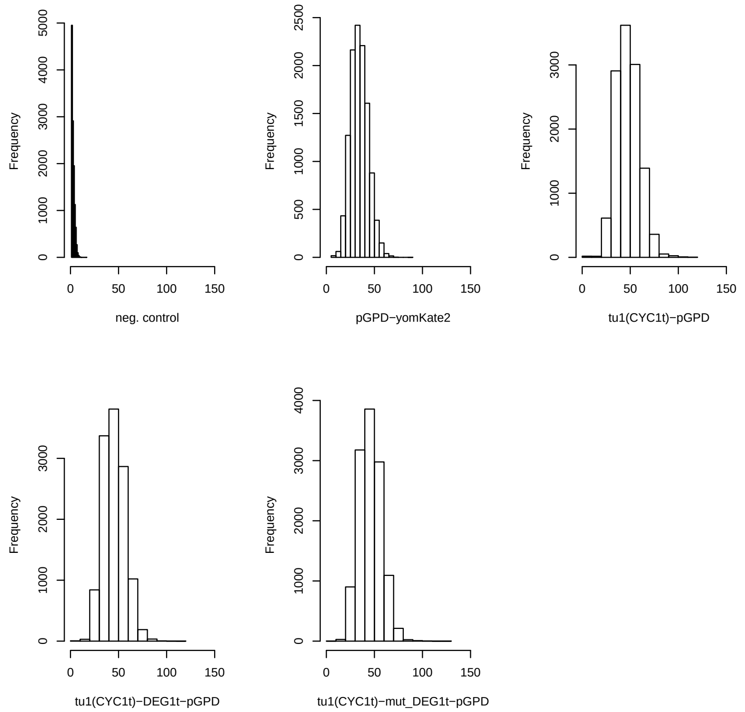

Figure S14: Red fluorescent protein expression by two-transcription-unit systems (see Figure S4 above). A) Box plots. Here, the contribution of yEGFP to the red fluorescence signal (about 14 AU) is not subtracted from the red fluorescence levels measured on the two-transcription-unit systems. B) Histograms.

A

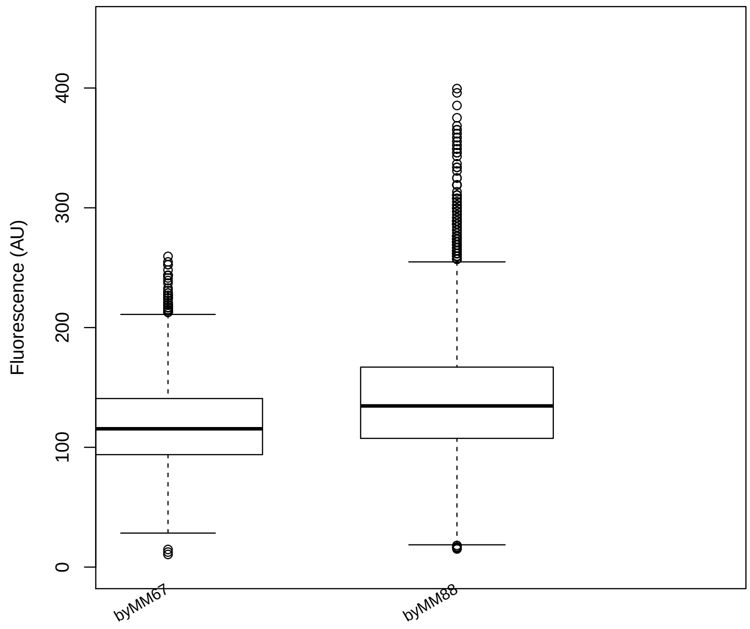

B

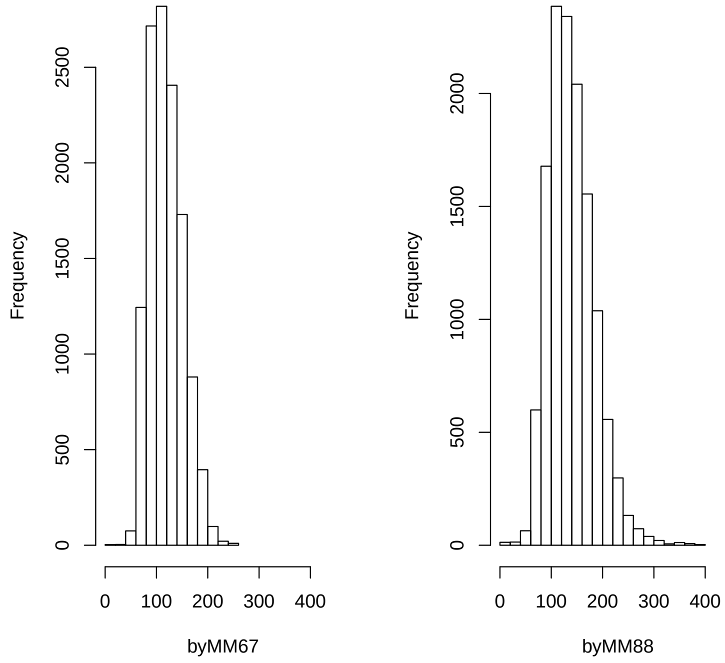

Figure S15: Comparison of fluorescence levels from plasmids assembled with different techniques (see Figure S5 above). A) Box plots. B) Histograms.

DNA part sequences

pGPD sequence

GAGCCCGCTTTTTAAGCTGGCATCCAGAAAAAAAAAAGAATCCCAGCACCAAAAT
ATTGTTTTCTTCACCAACCATCAGTTCATAGGTCCATTCTCTTAGCGCAACTACA
GAGAACAGGGGCACAAACAGGCAAAAAACGGGCACAACCTCAATGGAGTGAT
GCAACCTGCCTGGAGTAAATGATGACACAAGGCAATTGACCCACGCATGTATCT
ATCTCATTTTCTTACACCTTCTATTACCTTCTGCTCTCTCTGATTTGGAAAAAGCT
GAAAAAAAAAGGTTGAAACCAGTTCCCTGAAATTATTCCCCTACTTGACTAATAAG
TATATAAAGACGGTAGGTATTGATTGTAATTCTGTAAATCTATTTCTTAAACTTCTTA
AATTCTACTTTTATAGTTAGTCTTTTTTTTAGTTTTAAAACACCAAGAACTTAGTTTC
GAATAAACACACATAAACAAACAAAAAAAATGAGATAGATACATGCGTGGGTCAATT
GCCTTGTGTCATCATTTACTCCAGGCAGGTTGCATCACTCCATTGAGGTTGTGCC
CGTTTTTTGCCTGTTTGTGCCCCTGTTCTCTGTAGTTGCGCTAAGAGAATGGACC
TATGAACTGATGGTTGGTGAAGAAAACAATATTTTGGTGCTGGGATTCTTTTTTTTT
CTGGATGCCAGCTTAAAAAGCGGGCTC

mut_pGPD sequence

CAGTTCGAGTTTATCATTATCAATACTGCCATTTCAAAGAATACGTAAATAATTAATAG
TAGTGATTTTCCTAACTTTATTTAGTCAAAAAATTAGCCTTTTAATTCTGCTGTAACCC
GTACATGCCCAAAATAGGGGGCGGGTTACACAGAATATATAACATCGTAAGGTTTGA
CCGAGACAGTTTATTCCTGATTGGGGCTAAATATAATGGAGCCCGCTTTTTAAGCTG
GCATCCAGAAAAAAAAAAGAATCCCAGCACCAAAATATTGTTTTCTTCACCAACCATC
AGTTCATAGGTCCATTCTCTTAGCGCAACTACAGAGAACAGGGGCACAAACAGGCA
AAAAACGGGCACAACCTCAATGGAGTGATGCAACCTGCCTGGAGTAAATGATGACA
CAAGGCAATTGACCCACGCATGTATCTATCTCATTTTCTTACACCTTCTATTACCTTCT
GCTCTCTCTGATTTGGAAAAAGCTGAAAAAAAAAGGTTGAAACCAGTTCCCTGAAATT
ATTCCCCTACTTGACTAATAAGTATATAAAGACGGTAGGTATTGATTGTAATTCTGTAAA
TCTATTTCTTAAACTTCTTAAATTCTACTTTTATAGTTAGTCTTTTTTTTAGTTTTAAAACA
CCAAGAACTTAGTTTCGAATAAACACACATAAACAAACAAA

pTEF1 sequence

ATAGCTTCAAAATGTTTCTACTCCTTTTTTACTCTTCCAGATTTTCTCGGACTCCGCGCA
TCGCCGTACCACTTCAAAACACCCAAGCACAGCATACTAAATTTCCCCTCTTTCTTCCT
CTAGGGTGTCGTTAATTACCCGTACTAAAGGTTTGGAAAAGAAAAAAGAGACCGCCTC
GTTTCTTTTTCTTCGTCGAAAAAGGCAATAAAAATTTTTATCACGTTTCTTTTTCTTGAAA
ATTTTTTTTTTGATTTTTTTCTCTTTCGATGACCTCCCATTGATATTTAAGTTAATAAACGG
TCTTCAATTTCTCAAGTTTCAGTTTCATTTTTCTTGTTCTATTACAACTTTTTTTACTTCTT
GCTCATTAGAAAGAAAGCATAGCAATCTAATCTAAGTTT

pTEF2 sequence

GCTACCTATATTCCACCATAACATCAATCATGCGGTTGCTGGTGTATTTACCAATAATGTTT
AATGTATATATATATATATATATATGGGGCCGTATACTTACATATAGTAGATGTCAAGCGTAGGC
GCTTCCCCTGCCGGCTGTGAGGGCGCCATAACCAAGGTATCTATAGACCGCCAATCAG
CAAACTACCTCCGTACATTCATGTTGCACCCACACATTTATACACCCAGACCGCGACAAA
TTACCCATAAGGTTGTTTGTGACGGCGTCGTACAAGAGAACGTGGGAACTTTTTAGGCT
CACCAAAAAAGAAAGAAAAAATACGAGTTGCTGACAGAAGCCTCAAGAAAAAAAAAATT
CTTCTTCGACTATGCTGGAGGCAGAGATGATCGAGCCGGTAGTTAACTATATATAGCTAA
ATTGGTTCCATCACCTTCTTTTCTGGTGTCGCTCCTTCTAGTGCTATTTCTGGCTTTTCC
TATTTTTTTTTTTTCCATTTTTCTTTCTCTCTTTCTAATATATAAATTCTCTTGCATTTTCTATT

TTTCTCTCTATCTATTCTACTTGTTTATTCCCTTCAAGGTTTTTTTTTAAGGAGTACTTGTT
TTTAGAATATACGGTCAACGAACTATAATTAACTAAAC

pADH1 sequence

GGGTGTACAATATGGACTTCCTCTTTTCTGGCAACCAAACCCATACATCGGGATTCCT
ATAATACCTTCGTTGGTCTCCCTAACATGTAGGTGGCGGAGGGGAGATATACAATAGA
ACAGATACCAGACAAGACATAATGGGCTAAACAAGACTACACCAATTACACTGCCTCAT
TGATGGTGGTACATAACGAACTAATACTGTAGCCCTAGACTTGATAGCCATCATCATATC
GAAGTTTCACTACCCTTTTTCCATTTGCCATCTATTGAAGTAATAATAGGCGCATGCAAC
TTCTTTTCTTTTTTTTTCTTTTCTCTCTCCCCCGTTGTTGTCTCACCATATCCGCAATGAC
AAAAAAATGATGGAAGACACTAAAGGAAAAAATTAACGACAAAGACAGCACCAACAGAT
GTCGTTGTTCCAGAGCTGATGAGGGGTATCTCGAAGCACACGAAACTTTTTCCTTCCT
TCATTCACGCACACTACTCTCTAATGAGCAACGGTATACGGCCTTCCTTCCAGTTACTTG
AATTTGAAATAAAAAAAAGTTTGCTGTCTTGCTATCAAGTATAAATAGACCTGCAATTATTA
ATCTTTTGTTTCCTCGTCATTGTTCTCGTTCCCTTTCTTCCTTGTTTCTTTTTCTGCACAA
TATTTCAAGCTATACCAAGCATACAATCAACTATCTCATATACA

pACT1 sequence

CTTCCTTATCGGATCCTCAAAACCCTTAAAAACATATGCCTCACCCTAACATATTTTCCAAT
TAACCCTCAATATTTCTCTGTCACCCGGCCTCTATTTTCCATTTTCTTCTTTACCCGCCACG
CGTTTTTTTCTTTCAAATTTTTTTCTTCCTTCTTCTTTTTCTTCCACGTCCTCTTGCATAAATA
AATAAACCGTTTTGAAACCAAACTCGCCTCTCTCTCTCCTTTTTGAAATATTTTTGGGTTTGT
TTGATCCTTTCCTTCCCAATCTCTCTTGTTTAATATATATTCATTTATATCACGCTCTCTTTTTAT
CTTCCTTTTTTTCCTCTCTCTTGTATTCTTCCTTCCCCTTTCTACTCAAACCAAGAAGAAAAA
GAAAAGGTCAATCTTTGTTAAAGAATAGGATCTTCTACTACATCAGCTTTTAGATTTTTCACGC
TTACTGCTTTTTTTCTTCCCAAGATCGAAAATTTACTGAATTAACA

pCYC1min sequence

GCATGCATGTGCTCTGTATGTATATAAAACTCTTGTTTTCTTCTTTTCTCTAAATATTCTTT
CCTTATACATTAGGACCTTTGCAGCATAAATTACTATACTTCTATAGACACACAAACACAA
ATACACACACTAAATTAATA

pCYC1noTATA sequence

TTCTTTCCTTATACATTAGGACCTTTGCAGCATAAATTACTATACTTCTATAGACACA
CAAACACAAATACACACACTAAATTAATA

DEG1t sequence

AATAATATATAAACCTGTATAATATAACCTTGAAGACTATATTTCTTTTC

mut_DEG1t sequence

AATAAGAGATAAACCTGTATAATATAACCTTGAAGACTATATTTCTTTTC

Tsynth8 sequence

TATATAAACTCATTTACTTATGTAGGAATAAAGAGTATCATCTTTCAAA

mut_Tsynth8 sequence

GAGATAAACTCATTTACTTATGTAGGAATAAAGAGTATCATCTTTCAAA

genCYC1t sequence

ACAGGCCCCTTTTCCTTTGTCGATATCATGTAATTAGTTATGTCACGCTTACATTC
ACGCCCTCCTCCCACATCCGCTCTAACCGAAAAGGAAGGAGTTAGACAACCTG
AAGTCTAGGTCCCTATTTATTTTTTTTAATAGTTATGTTAGTATTAAGAACGTTATTT
ATATTTCAAATTTTTCTTTTTTTTCTGTACAAACGCGTGTACGCATGTAACATTATA

CYC1t sequence

CATGTAATTAGTTATGTCACGCTTACATTCACGCCCTCCCCCCACATCCGCTCTA
ACCGAAAAGGAAGGAGTTAGACAACCTGAAGTCTAGGTCCCTATTTATTTTTTTA
TAGTTATGTTAGTATTAAGAACGTTATTTATATTTCAAATTTTTCTTTTTTTTCTGTA
CAGACGCGTGTACGCATGTAACATTATACTGAAAACCTTGCTTGAGAAGGTTTTG
GGACGCTCGAAGGCTTTAATTTGCAAGCT

shortADH1t sequence

CTAATAAGTTATAAAAAAAATAAGTGTATACAAATTTTAAAGTGACTCTTAGGTTTTA
AAACGAAAATTCTTATTCTTGAGTAACTCTTTCCTGTAGGTCAGGTTGCTTTCTCA
GGTATAGCATGAGGTCGCTCTTATTGACCACACCTCTACCGGCATG

s100 sequence

GAATTATTTGATCACCAAGGTGCAGAGCCAGCCTTCTTATTCGGCCTTGAATTGA
TCATATGCGGATTAGAAAAACAACTTAAATGTGAAAGTGGATCTT

yEGFP sequence

ATGTCTAAAGGTGAAGAATTATTCACTGGTGTTGTCCCAATTTTGGTTGAATTAGA
TGGTGATGTTAATGGTCACAAATTTTCTGTCTCCGGTGAAGGTGAAGGTGATGCT
ACTTACGGTAAATTGACCTTAAAATTTATTTGTACTACTGGTAAATTGCCAGTTCCA
TGGCCAACCTTAGTCACTACTTTCGGTTATGGTGTTCAATGTTTTGCGAGATACCC
AGATCATATGAAACAACATGACTTTTTCAAGTCTGCCATGCCAGAAGGTTATGTTCA
AGAAAGAACTATTTTTTTCAAAGATGACGGTAACTACAAGACCAGAGCTGAAGTCA
AGTTTGAAGGTGATACCTTAGTTAATAGAATCGAATTAAAAGGTATTGATTTTAAAGA
AGATGGTAACATTTTAGGTCACAAATTGGAATACAACTATAACTCTCACAATGTTTAC
ATCATGGCTGACAAACAAAAGAATGGTATCAAAGTTAACTTCAAAATTAGACACAACA
TTGAAGATGGTTCTGTTCAATTAGCTGACCATTATCAACAAAATACTCCAATTGGTGA
TGGTCCAGTCTTGTTACCAGACAACCATTACTTATCCACTCAATCTGCCTTATCCAAA
GATCCAAACGAAAAGAGAGACCACATGGTCTTGTTAGAATTTGTTACTGCTGCTGGT
ATTACCCATGGTATGGATGAATTGTACAAATAA

yEGFPgg sequence

ATGTCTAAAGGTGAAGAATTATTCACTGGTGTTGTCCCAATTTTGGTTGAATTAGATG
GTGATGTTAATGGTCACAAATTTTCTGTCTCCGGTGAAGGTGAAGGTGATGCTACTTA
CGGTAAATTGACCTTAAAATTTATTTGTACTACTGGTAAATTGCCAGTTCCATGGCCAA
CCTTAGTCACTACTTTCGGTTATGGTGTTCAATGTTTTGCGAGATACCCAGATCATAT
GAAACAACATGACTTTTTCAAGTCTGCCATGCCAGAAGGTTATGTTCAAGAAAGAAC
TATTTTTTTCAAAGATGACGGTAACTACAAGACCAGAGCTGAAGTCAAGTTTGAAGG
TGATACCTTAGTTAATAGAATCGAATTAAAAGGTATTGATTTTAAAGAAGATGGTAACA
TTTTAGGTCACAAATTGGAATACAACTATAACTCTCACAATGTTTACATCATGGCTGAC
AAACAAAAGAATGGTATCAAAGTTAACTTCAAAATTAGACACAACATTGAAGATGGTTC
TGTTCAATTAGCTGACCATTATCAACAAAATACTCCAATTGGTGATGGTCCAGTCTTGT
TACCAGACAACCATTACTTATCCACTCAATCTGCCTTATCCAAAGATCCAAACGAAAAG

AGGGACCACATGGTCTTGTTAGAATTTGTTACTGCTGCTGGTATTACCCATGGTATGGA
TGAATTGTACAAATAA

yomKate2 sequence

ATGGTTTCTGAACTCATCAAGGAAAACATGCACATGAAACTTTACATGGAAGGTACTGT
GAACAATCATCATTTTAAGTGTACATCCGAGGGTGAAGGCAAACCTTACGAAGGAACTC
AAACTATGAGAATTAAAGCTGTAGAAGGTGGACCATTACCTTTTGCATTTGATATCTTGG
CAACATCATTCATGTATGGGAGCAAGACATTCATAAACCATACTCAAGGTATACCAGACTT
TTTCAAACAGAGTTTTCCAGAGGGTTTTACATGGGAAAGAGTAACAACGTACGAGGATGG
AGGTGTATTGACAGCCACTCAAGACACATCACTTCAAGATGGGTGTTTAATCTACAATGT
CAAGATTAGAGGCGTCAATTTCCCTTCTAATGGTCCAGTTATGCAGAAAAAGACATTAGG
CTGGGAAGCGTCAACCGAAACCCTGTACCCTGCTGATGGTGGCCTAGAAGGCAGAGCT
GACATGGCCCTTAAACTGGTTGGTGGAGGGCATCTAATCTGCAATTTGAAAACCACTTATC
GTTCTAAAAAGCCAGCCAAAAACCTAAAGATGCCAGGTGTTTACTACGTCGACCGAAGATT
AGAAAGGATTAAAGAGGCTGATAAAGAGACTTATGTTGAACAACACGAAGTGGCAGTGGC
TAGATACTGTGATTTGCCATCTAAGTTGGGACACAGATAA

References

[1] Marchisio MA: **In silico design and in vivo implementation of yeast gene Boolean gates.** *Journal of Biological Engineering* 2014, **8**:6.

[2] Sherman F: **Getting Started with Yeast**. *Methods Enzymol.* 2002, **350**:3–41.

[3] Marchisio MA, Colaiacovo M, Whitehead E, Stelling J: **Modular, rule-based modeling for the design of eukaryotic synthetic gene circuits.** *BMC Systems Biology* 2013, **7**:42.

[4] Hoops S, Sahle S, Gauges R, Lee C, Pahle J, Simus N, Singhal M, Xu L, Mendes P, Kummer U: **COPASI–a COmplex PAthway SImulator.** *Bioinformatics* 2006, **22**(24):3067–3074.
